# Metabolic Versatility of the Family *Halieaceae* Revealed by the Genomics of Novel Cultured Isolates

**DOI:** 10.1128/spectrum.03879-22

**Published:** 2023-03-14

**Authors:** Shan-Hui Li, Ilnam Kang, Jang-Cheon Cho

**Affiliations:** a Department of Biological Sciences and Bioengineering, Inha University, Incheon, Republic of Korea; b Center for Molecular and Cell Biology, Inha University, Incheon, Republic of Korea; Swansea University

**Keywords:** coastal seas, cultivation, phototrophy, genomics, metabolic capacity, phytoplankton blooms, *Halieaceae*

## Abstract

The family *Halieaceae* (OM60/NOR5 clade) is a gammaproteobacterial group abundant and cosmopolitan in coastal seawaters and plays an important role in response to phytoplankton blooms. However, the ecophysiology of this family remains understudied because of the vast gap between phylogenetic diversity and cultured representatives. Here, using six pure cultured strains isolated from coastal seawaters, we performed in-depth genomic analyses to provide an overview of the phylogeny and metabolic capabilities of this family. The combined analyses of 16S rRNA genes, genome sequences, and functional genes relevant to taxonomy demonstrated that each strain represents a novel species. Notably, two strains belonged to the hitherto-uncultured NOR5-4 and NOR5-12 subclades. Metabolic reconstructions revealed that the six strains likely have aerobic chemo- or photoheterotrophic lifestyles; five of them possess genes for proteorhodopsin or aerobic anoxygenic phototrophy. The presence of blue- or green-tuned proteorhodopsin in *Halieaceae* suggested their ability to adapt to light conditions varying with depth or coastal-to-open ocean transition. In addition to the genes of anaplerotic CO_2_ fixation, genes encoding a complete reductive glycine pathway for CO_2_ fixation were found in three strains. Putative polysaccharide utilization loci were detected in three strains, suggesting the association with phytoplankton blooms. Read mapping of various metagenomes and metatranscriptomes showed that the six strains are widely distributed and transcriptionally active in marine environments. Overall, the six strains genomically characterized in this study expand the phylogenetic and metabolic diversity of *Halieaceae* and likely serve as a culture resource for investigating the ecophysiological features of this environmentally relevant bacterial group.

**IMPORTANCE** Although the family *Halieaceae* (OM60/NOR5 clade) is an abundant and cosmopolitan clade widely found in coastal seas and involved in interactions with phytoplankton, a limited number of cultured isolates are available. In this study, we isolated six pure cultured *Halieaceae* strains from coastal seawaters and performed a comparative physiological and genomic analysis to give insights into the phylogeny and metabolic potential of this family. The cultured strains exhibited diverse metabolic potential by harboring genes for anaplerotic CO_2_ fixation, proteorhodopsin, and aerobic anoxygenic phototrophy. Polysaccharide utilization loci detected in some of these strains also indicated an association with phytoplankton blooms. The cultivation of novel strains of *Halieaceae* and their genomic characteristics largely expanded the phylogenetic and metabolic diversity, which is important for future ecophysiological studies.

## INTRODUCTION

The family *Halieaceae* belonging to the class *Gammaproteobacteria* has been proposed to accommodate the NOR5/OM60 clade of the oligotrophic marine *Gammaproteobacteria* (OMG) group (1, 2). This family comprises 13 subclades widely distributed in various environments, including ocean surface, deep-sea sediment, freshwater, saline lakes, and soil. The family is known to be abundant in coastal surface waters (up to ~11% of the total bacterioplankton community) (1, 3, 4), especially in the North Sea during diatom-dominated phytoplankton blooms (up to ~13%) (5). Several isolates have been allocated to this family (2); however, most subclades of the family still lack cultured representatives (3). Although bypassing the isolation of uncultivated strains via metagenomics and single-cell genomics has proven to be an asset for studying not-yet-cultivated microbes, cultivation remains a reliable approach to characterize a specific group of microbes in detail and gain definitive information on its phylogenetic, metabolic, and ecological relevance (6). Moreover, functional predictions based on omics largely rely on well-annotated genomes from cultured representatives (7). Thus, culturing remains necessary to marine microbial ecology, as only a small minority of microorganisms are currently cultivated (8, 9).

With an increased number of cultured isolates and genome sequences available in *Halieaceae*, diverse metabolic potentials have been found. *Halieaceae* members were originally considered obligate oligotrophs because of the lack of their colony-forming ability (1). Since then, a typical *K*-strategist has been proposed as the trophic strategy of this heterotrophic lineage based on genome-wide gene content analyses (2). Some members are characterized by cyanophycin production (10) and propylene assimilation (11). In addition, some are predicted to be photoheterotrophs by using aerobic anoxygenic phototrophy (AAP) (4, 12, 13) or by possessing proteorhodopsin (PR) (14). Many AAP members belong to *Halieaceae*, with a demonstration of light-enhanced growth for several strains (13), whereas PR-containing strains have been rarely found in the family, which is different from those in studies that show the predominance of rhodopsin over AAP in oceanic bacteria (15–17). A recent metagenomic study has revealed that a marine alphaproteobacterial group has genes for AAP, sulfur compound oxidation, and carbon fixation, implicating primary production (18). Some AAP-equipped *Halieaceae* strains also encode the Sox enzyme complex for thiosulfate oxidation (19); however, only genes for anaplerotic reactions to fix carbon have been reported (14, 20). Culturing AAP-using and PR-containing microbes and deciphering their genomes can provide better insights into their ecophysiology in the ocean.

A phytoplankton bloom triggers a massive release of polysaccharides that leads to the succession of specialized bacterial clades that can rapidly incorporate alga-derived dissolved organic matter into biomass (21, 22). Decomposition is catalyzed by carbohydrate-active enzymes (CAZymes) classified into several categories, i.e., glycoside hydrolase (GH), polysaccharide lyase (PL), carbohydrate esterase (CE), glycosyltransferase (GT), carbohydrate-binding module (CBM), and enzymes with auxiliary activities (AA) (23). Polysaccharide utilization loci (PULs) found in members of *Flavobacteriia* of the *Bacteroidetes* can bind to, degrade, and transport complex carbohydrates (24). The canonical *Bacteroidetes* PULs contain tandem *susC* and *susD* homologs encoding TonB-dependent transporters (TBDTs) and glycan-binding lipoprotein, respectively (24). Although *Gammaproteobacteria* lacks *susC*-*susD* pairs, the hallmark of *Bacteroidetes* PULs, degradation systems analogous to *Bacteroidetes* PULs exist (24). Notably, the genomic and physiological features of *Gammaproteobacteria* have not yet been studied at the same level of detail as other dominant heterotrophic bacterial responders, i.e., *Flavobacteriia* and *Alphaproteobacteria* (5, 25). Considering the recurrent occurrence of *Halieaceae* during phytoplankton blooms in the North Sea (22), studies of their genomes will help elucidate the dynamics of *Halieaceae* during coastal blooming.

Here, we report the characteristics of six strains of *Halieaceae*, sporadically isolated from coastal seas around Korea. Their genomes were sequenced and compared to obtain insights into their phylogeny, metabolism, and ecological features. These strains were taxonomically proposed to constitute novel species or genera, and two strains were the first representatives of the uncultured NOR5-4 and NOR5-12 subclades. Their versatile metabolism helps us better understand the ecological success of *Halieaceae* in the ocean.

## RESULTS AND DISCUSSION

### Strain isolation, revival, and cultivation.

More than 60 strains of *Halieaceae* were isolated from coastal surface seawaters of the Korean Peninsula in 2005 to 2017 by using several cultivation media and techniques (see Table S1 and Fig. S1 in the supplemental material). These putative *Halieaceae* axenic cultures were revived from glycerol stocks and examined for growth sustainability and culture purity upon repeated transfer in the laboratory to obtain actively and robustly growing pure cultures. Six pure cultured strains belonging to different lineages were selected for whole-genome sequencing to expand the genomic coverage of *Halieaceae* (Fig. S2). Among them, five strains (IMCC8485, IMCC11814, IMCC14385, IMCC14734, and JH123) were isolated using a high-throughput culturing (HTC) technique with different medium constituents from the East Sea of Korea, and IMCC3088 was isolated on an agar plate by using a standard dilution technique from the Yellow Sea. IMCC3088 and IMCC14385 formed colonies on semisolid agar media, whereas the other four strains did not grow on any agar medium (IMCC8485, IMCC11814, and JH123) or formed small colonies only sporadically (IMCC14734), possibly indicating their oligotrophic lifestyle. Oligotrophy has been considered a crucial factor leading to the fastidious growth of environmental microorganisms in a laboratory (26), often accompanied by an inability to form colonies. In this respect, these results showed that both the colony formers and nonformers of *Halieaceae* were cultivated successfully using the HTC method.

### General genomic characteristics.

**FIG 1:**
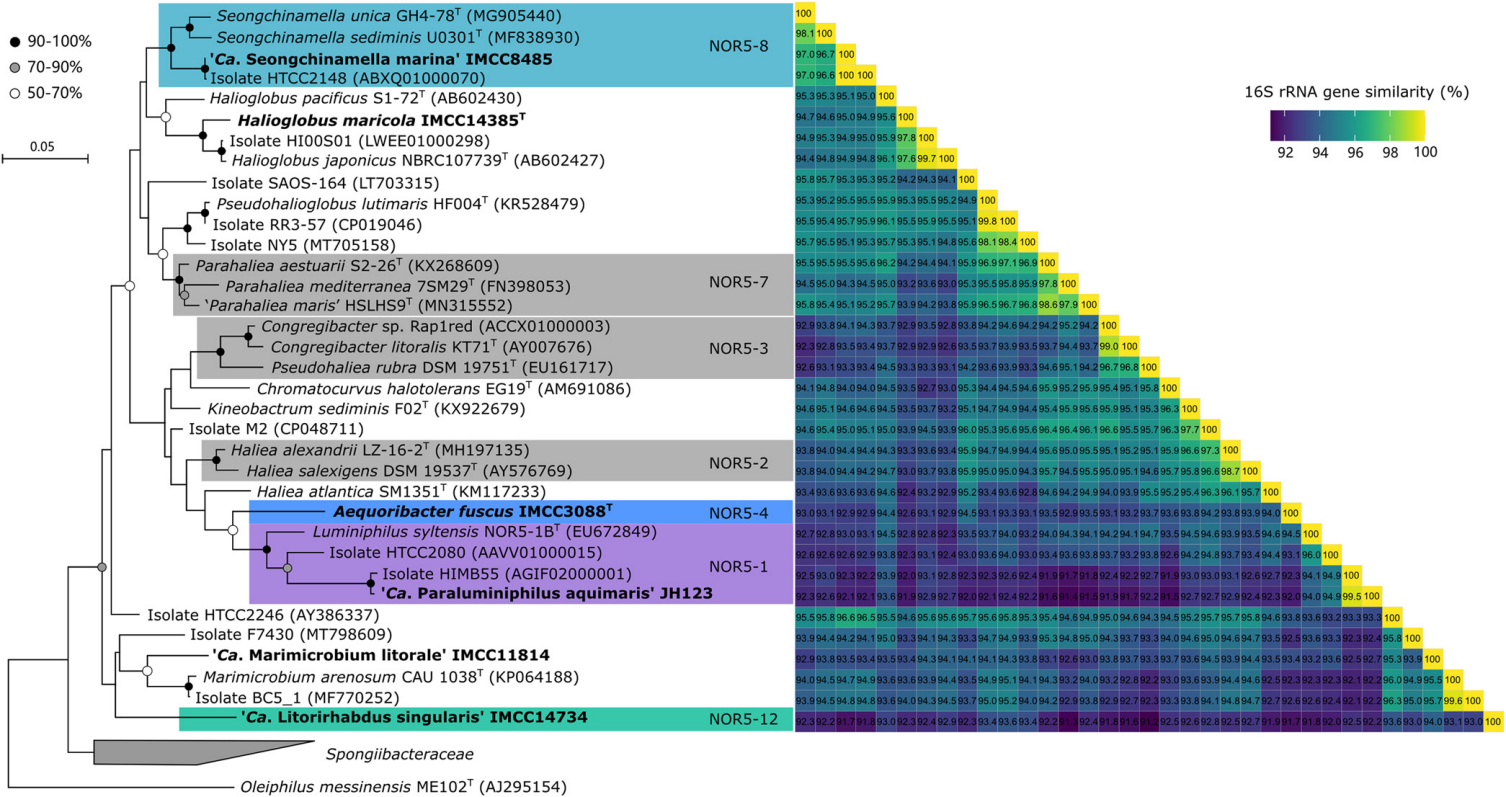
Phylogenetic tree of 16S rRNA gene sequences from the six strains in this study and closely related cultured *Halieaceae* members. The six strains in this study are highlighted in bold. The 16S rRNA gene sequence from Oleiphilus messinensis ME102^T^ (GenBank accession no. AJ295154) was selected as an outgroup. The phylogeny shown here was generated using RAxML (version 8.2.7). Bootstrap values (≥50%; 100 replicates) are indicated on nodes based on the legend at the upper left. Bar, 0.05 substitution per nucleotide position.

**FIG 2:**
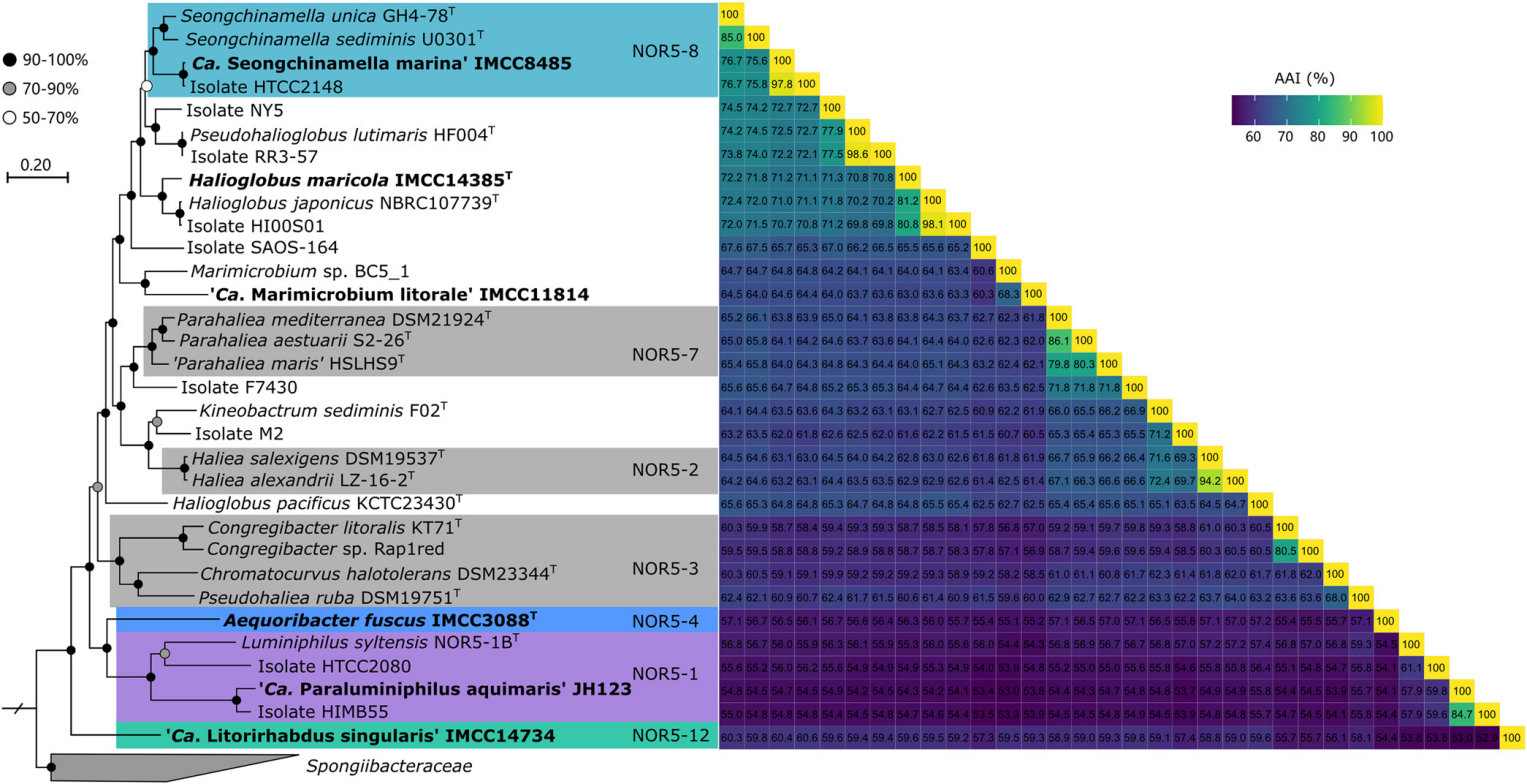
Phylogenomic tree of the six strains in this study and closely related cultured *Halieaceae* members. The maximum likelihood tree was constructed using RAxML (version 8.2.7) based on a concatenated alignment of 92 single-copy core genes. The Oleiphilus messinensis ME102^T^ (GenBank accession no. CP021425) genome was used as an outgroup. The NOR5 subclades proposed by Yan et al. (3) are indicated by color-shaded rectangles. Bootstrap support values (≥50%; 100 replicates) are indicated on nodes based on the legend at the upper left. The scale bar represents 20% estimated sequence divergence. The heat map on the right shows the AAI values among the 32 genomes of the cultured *Halieaceae* members included in the analysis.

We successfully obtained the genome sequences of these six pure cultured strains. Two genomes (IMCC3088 and IMCC14385) sequenced by using the PacBio platform were assembled into a single circular contig, and the assembly of four genomes (IMCC8485, IMCC11814, IMCC14734, and JH123) obtained using the Illumina platform comprised 1 to 19 linear contigs. The general genomic features of the six strains in this study are summarized in Table 1. The six *Halieaceae* genomes showed rather large differences in genome size, ranging from 2.7 Mbp to 4.8 Mbp. The smallest genome (~2.7 Mbp) of JH123 might suggest a reductive evolution and genome streamlining typical of oligotrophic free-living bacteria (27). This difference in genome size likely reflects the phylogenetic positions within *Halieaceae*, since the genome sizes of JH123, IMCC8485, and IMCC14385 were found to be similar to those of closely related strains (Fig. 1 and 2). The DNA GC content of the genomes ranged from 51.7% to 56.7% (Table 1). The two small genomes (JH123 and IMCC3088) showed the lowest GC contents, but an overall correlation between genome size and GC content was not evident. Genome-scale similarity analysis among the six strains is presented in Fig. S3. The complete genomes of IMCC3088 and IMCC14385 had two complete rRNA operons comprising 16S, 23S, and 5S rRNA genes. JH123 consists of a single contig, harboring only one rRNA operon. For the three other genomes consisting of 6 to 19 contigs, only one copy of full-length 5S, 16S, and 23S rRNA genes was predicted. However, the sequencing depths of the contigs from which rRNA genes were annotated were ~2-fold higher than the average sequencing depth of the respective genomes for all three strains, indicating that the copy number of rRNA genes in these genomes is highly likely to be two. The two 16S rRNA genes of IMCC3088 had the same length of 1,536 bp but were not identical to each other (99.4% identity); their differences (9 bp) were mainly found in a short region (~20 bp) corresponding to helix H17, which was reported to have intragenomic heterogeneity in *Vibrio* strains (28, 29).

**TABLE 1:** General genomic features of the six strains in this study

Feature	“*Ca*. P. aquimaris” JH123	*A. fuscus* IMCC3088	“*Ca*. S. marina” IMCC8485	*H. maricola* IMCC14385	“*Ca*. M. litorale” IMCC11814	“*Ca*. L. singularis” IMCC14734
IMG/MER ID	2765235711	2739368038	2744055030	2739368037	2744055039	2744055040
Genome size (kbp)	2,724.4	30,95.4	4,355.5	4,306.4	4,074.3	4,834.0
Contigs	1	1	19	1	6	17
GC content (%)	52.7	51.7	52.9	56.7	54.6	54.2
Total no. of genes	2,512	2,866	3,911	3,943	3,688	4,377
No. of protein coding genes	2,463	2,813	3,859	3,888	3,639	4,328
No. of RNA genes	49	53	52	55	49	49
No. of rRNA genes	3	6	4	6	3	4
5S rRNA	1	2	2	2	1	1
16S rRNA	1	2	1	2	1	1
23S rRNA	1	2	1	2	1	2
No. of tRNA genes	40	41	40	42	42	39
No. of genes with signal peptides	226	284	371	513	356	483
No. of genes with transmembrane helices	546	593	846	903	842	1,029

The six genomes had large numbers of genes encoding signal peptides and transmembrane helices, ranging from 226 to 513 and from 546 to 1029, respectively. The wide ranges might indicate differences in the repertoire of membrane-bound and periplasmic proteins, putatively leading to the varied transport of growth substances in the cell membrane. A CRISPR-Cas locus belonging to the type II system was predicted in IMCC8485, suggesting that it might be subject to phage infection in the marine environment. The six spacer sequences (29 to 30 bp) predicted in the CRISPR array of IMCC8485 were analyzed by BLASTn against the DNA database in the Virus BLAST of IMG. One of the spacers showed an exact match (100%; 29 bp) to a scaffold (12.3 kb) assembled from a viral metagenome of the Delaware Bay.

### Taxonomic classification and proposal.

Phylogenetic trees based on 16S rRNA gene sequences showed that the six strains in this study were widely distributed within *Halieaceae*, with four strains belonging to different subclades (NOR5-1, NOR5-4, NOR5-8, and NOR5-12) proposed by Yan et al. (3) and two strains not clearly affiliated with previously proposed subclades (Fig. 1). Phylogenomic trees based on all cultured *Halieaceae* genomes showed similar tree topologies (Fig. 2). In addition to these phylogenetic analyses, the taxonomic assignment (presented in the next paragraph) was inferred on the basis of 16S rRNA gene sequence similarity (98.7% and 94.5% for species and genus demarcation, respectively) (30, 31), average nucleotide identity (ANI; 95 to 96% and 74.0% for species and genus demarcation, respectively) (32, 33), average amino acid identity (AAI; 60% for genus demarcation) (34), alignment fraction (AF; 33.1% for genus demarcation) (33), percentage of conserved proteins (POCP; 50% for genus demarcation) (35), Genome Taxonomy Database Toolkit (GTDB-Tk) classification (36), and presence or absence of genes related to the biosynthesis of polar lipids, one of the important biochemical markers in bacterial taxonomy.

Analyses of 16S rRNA gene sequence similarities, ANI, and AAI suggested that the six strains belonged to different genera or species of *Halieaceae* (Fig. 1 and 2). The taxonomic assignment of IMCC3088 as Aequoribacter fuscus gen. nov., sp. nov. (14), and IMCC14385 as Halioglobus maricola sp. nov. (37) was reported using a polyphasic approach in our previous studies. JH123 formed a robust cluster with HIMB55, HTCC2080, and Luminiphilus syltensis NOR5-1B^T^ in 16S rRNA gene- and genome-based trees. It shared the highest 16S rRNA gene sequence identity with HIMB55 (99.5%) of the NOR5-1C subclade and with HTCC2080 (94.9%) and *L. syltensis* NOR5-1B^T^ (94.0%) of the NOR5-1B subclade. It had an ANI of 68.0%, AF of 24.5%, AAI of 57.9%, and POCP of 52.7% with *L. syltensis* NOR5-1B^T^. Except for the POCP (52.7%) value, which was slightly higher than the threshold for genus demarcation (50%), all other molecular details indicated that JH123 belonged to a novel genus. *L. syltensis* NOR5-1B^T^ contained all genes associated with the synthesis of diphosphatidylglycerol (DPG), phosphatidylglycerol (PG), and phosphatidylethanolamine (PE), but the gene related to DPG biosynthesis was absent in the JH123 genome, suggesting the designation of JH123 as a novel genus. Thus, we propose “*Candidatus* Paraluminiphilus aquimaris” gen. nov., sp. nov., for JH123.

IMCC8485 robustly clustered with HTCC2148 and *Seongchinamella* species in both trees. It shared the highest 16S rRNA gene sequence identity with HTCC2148 (100%) of NOR5-8, *Seongchinamella unica* GH4-78^T^ (97.0%), and *Seongchinamella sediminis* U0301^T^ (96.7%). ANI, AF, AAI, and POCP values between IMCC8485 and the two *Seongchinamella* species were 74.6 to 74.7%, 50.4 to 55.1%, 75.6 to 76.7%, and 65.5 to 70.1%, respectively. IMCC8485 contained all genes associated with the synthesis of DPG, PG, and PE, which were detected in *Seongchinamella* species. All results suggested that IMCC8485 would be a novel species of *Seongchinamella.* Thus, we propose IMCC8485 as “*Candidatus* Seongchinamella marina” sp. nov.

Strain IMCC11814 was clustered with BC5_1 and Marimicrobium arenosum CAU 1038^T^ without strong bootstrap support (≤70%) in the 16S rRNA gene-based tree, but it robustly clustered with BC5_1 in the genome-based tree (the *M. arenosum* CAU 1038^T^ genome was unavailable in public databases). IMCC11814 shared the highest 16S rRNA gene sequence similarity with BC5_1 (95.7%) and *M. arenosum* CAU 1038^T^ (95.5%), suggesting that it represents a different species of *Marimicrobium*. AAI and POCP between IMCC11814 and BC5_1 (*Marimicrobium* sp.) were 68.3% and 62.1%, respectively, suggesting that they belonged to the same genus. IMCC11814 and BC5_1 contained all genes associated with the synthesis of DPG, PG, and PE. Thus, we propose IMCC11814 as “*Candidatus* Marimicrobium litorale” sp. nov.

IMCC14734 of the NOR5-12 subclade formed a distinct clade in the 16S rRNA gene tree and genomic tree that was not associated with any other known genera. Moreover, IMCC14734 shared ≤94.0% 16S rRNA gene sequence similarities with known cultured isolates, suggesting that it could be a novel genus. ANI, AF, AAI, and POCP between IMCC14734 and IMCC11814 were 68.4%, 23.9%, 59.3%, and 58.4%, respectively; ANI, AF, AAI, and POCP between IMCC14734 and *P. lutimaris* HF004^T^ were 69.4%, 24.1%, 59.4%, and 53.8%, respectively. Except for the POCP, other molecular findings indicated that IMCC14734 belonged to a novel genus, which was also consistent with the GTDB-Tk classification. Furthermore, all genes related to the synthesis of DPG, PG, and PE were identified in the “*Ca.* Marimicrobium litorale” sp. nov. strain IMCC11814 and *P. lutimaris* HF004^T^ genomes, but the gene encoding phosphatidylglycerophosphatase related to PG and DPG synthesis was not annotated in IMCC14734. Thus, we propose IMCC14734 as “*Candidatus* Litorirhabdus singularis” gen. nov., sp. nov.

### Central carbon metabolism.

**FIG 3:**
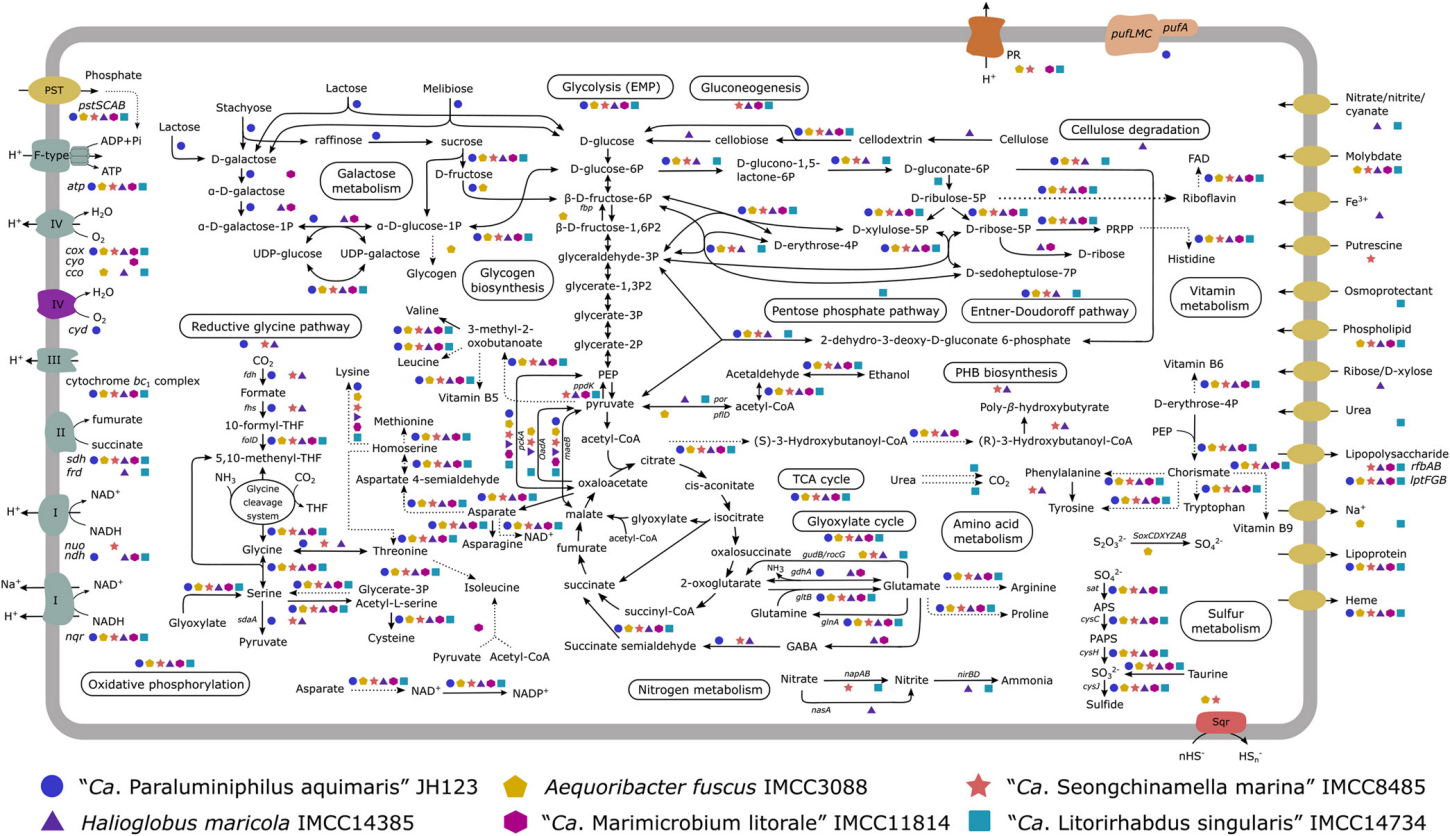
Overview of the metabolic potential of the six strains in this study inferred from the genome sequences. The presence of metabolic steps is indicated by colored symbols representing the genomes based on the legend at the bottom. Some major metabolic modules/pathways are indicated by rounded boxes in the cell diagram. Dashed lines indicate reactions involving two or more reaction steps. PR, proteorhodopsin; PEP, phosphoenolpyruvate; PRPP, 5-phospho-α-d-ribose 1-diphosphate; EMP, Embden-Meyerhof-Parnas pathway; PHB, poly-β-hydroxybutyrate.

Metabolic reconstruction based on genome annotation suggested the overall heterotrophic lifestyle of the six strains (Fig. 3). The six strains harbored the complete Embden-Meyerhof-Parnas pathway of glycolysis, the tricarboxylic acid cycle (TCA), and the glyoxylate cycle but differed in the Entner-Doudoroff pathway and the pentose phosphate pathway (PPP). The complete Entner-Doudoroff pathway was found in all strains except for “*Ca.* M. litorale” IMCC11814. Although all strains harbored the nonoxidative branch of PPP, the oxidative branch was present only in “*Ca.* L. singularis” IMCC14734. Gluconeogenesis was not complete in any strains, but four strains (“*Ca.* S. marina” IMCC8485, *H. maricola* IMCC14385, “*Ca.* M. litorale” IMCC11814, and “*Ca.* L. singularis” IMCC14734) possessed the genes for pyruvate orthophosphate dikinase (*ppdK*) that would allow the conversion of pyruvate to phosphoenolpyruvate (PEP). A gene for fructose-1,6-bisphosphatase (*fbp*) to convert β-d-fructose 1,6-bisphosphate to β-d-fructose 6-phosphate was found in only *A. fuscus* IMCC3088. In the TCA cycle, in addition to the 2-oxoglutarate dehydrogenase complex, genes encoding 2-oxoglutarate/2-oxoacid ferredoxin oxidoreductase for converting 2-oxoglutarate to succinyl coenzyme A were found in all genomes, except for “*Ca*. P. aquimaris” JH123. This enzyme, generally expressed by strictly anaerobic and microaerophilic organisms, is often found to operate under aerobic conditions (38). A detailed description of the metabolism of other carbohydrates is provided in the supplemental material.

### Aerobic respiration.

The presence of the complete gene sets of oxidative phosphorylation and F-type ATPase for ATP production in the six strains suggested the capacity to respire oxygen. Regarding terminal oxidases, the six strains contained genes encoding *aa_3_*-type cytochrome *c* oxidase (*coxABC*). In addition, genes for *cbb_3_*-type cytochrome *c* oxidase (*ccoNOQP*) were detected in *A. fuscus* IMCC3088, *H. maricola* IMCC14385, and “*Ca.* L. singularis” IMCC14734. Two quinol oxidases, cytochrome *bo_3_* oxidase (*cyoABCD*) and cytochrome *bd* oxidase (*cydAB*), were detected in “*Ca.* M. litorale” IMCC11814 and “*Ca*. P. aquimaris” JH123, respectively. It is known that *cbb_3_*- and *bd*-type oxidases show a higher oxygen affinity than *aa_3_* and *bo_3_* type oxidases (39). Thus, the redundant terminal oxidase might reflect respiratory flexibility in the environment. A detailed description of other components of aerobic respiration is provided in the supplemental material.

### Light utilization and phototrophy.

**FIG 4:**
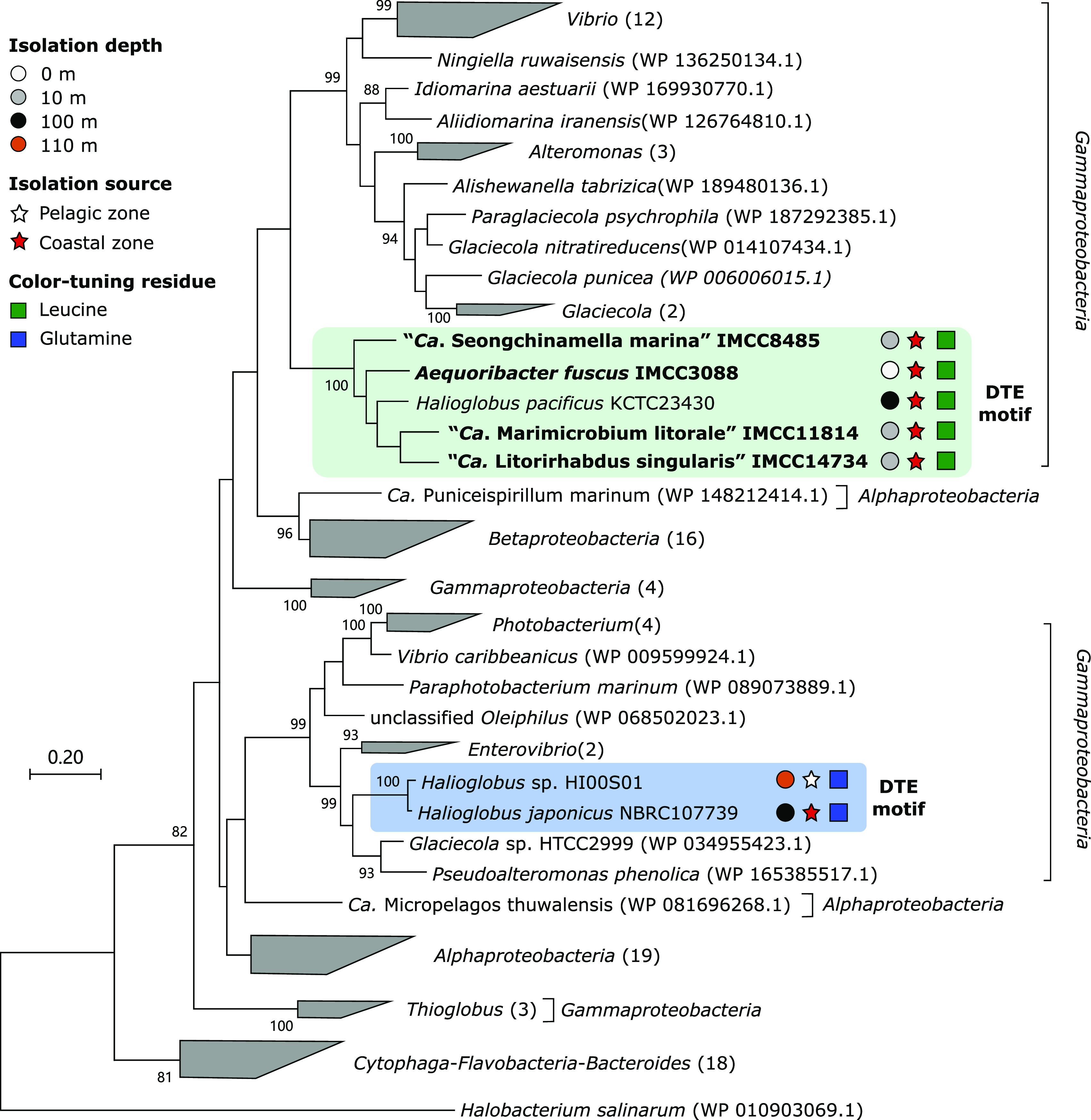
Phylogenetic position of rhodopsin sequences found in *Halieaceae* strains. The rhodopsins encoded in the cultured *Halieaceae* strains are indicated by colored backgrounds; names in bold type indicate the strains in this study. For *Halieaceae* rhodopsins, isolation sources and depths of the strain and the amino acid residue at the color-tuning position are indicated at the right of strain names based on the legends at the upper left. Phylogenetic affiliation at the class level is indicated by brackets. When necessary for visualization, sequences from the same class/genus were grouped as indicated by shaded wedges, followed by the number of grouped sequences in parentheses. The amino acid sequences were aligned using MUSCLE (version 3.8). The phylogenetic tree was constructed using the maximum likelihood method in MEGA X (version 10.1). Bootstrap values (≥70%; 100 replicates) are indicated above the branches. The scale bar displays the number of substitutions per amino acid.

Five strains had genes for light utilization based on PRs or AAP. *A. fuscus* IMCC3088, “*Ca.* S. marina” IMCC8485, “*Ca.* M. litorale” IMCC11814, and “*Ca.* L. singularis” IMCC14734 encoded light-harvesting proton-pumping PRs, and “*Ca*. P. aquimaris” JH123 had a gene cluster for AAP; *H. maricola* IMCC14385 had neither of them. Regarding the rhodopsin genes found in the four strains, phylogenetic analyses demonstrated that all of them are proton-pumping PRs with a DTE motif (Fig. 4; Fig. S4) (40). The *Halieaceae* PRs fell into two phylogenetically distinct clades within a large radiation of bacterial PRs. PRs in the four strains of this study formed a monophyletic lineage, together with the PR of Halioglobus pacificus KCTC23430^T^, while they were separated from PRs in other *Halieaceae* members, i.e., *Halioglobus* sp. HI00S01 and Halioglobus japonicus NBRC107739^T^ (Fig. 4). The two groups of PRs were different in terms of spectral tuning. PRs of the four strains and *H. pacificus* KCTC23430^T^, all of which were isolated from depths of less than 100 m in coastal seas, contained leucine at position 105, suggesting tuning to green (or yellow) light in the ocean surface (41–43). Conversely, the PRs of two *Halioglobus* species, HI00S01 (isolated from a depth of 110 m of open ocean) and *H. japonicus* NBRC107739^T^ (from a depth of 100 m of the coastal sea) contained glutamine at the corresponding position, typical for blue-absorbing PRs usually detected in the open ocean and deeper seawaters (41, 43). Thus, the color-tuning variants of PR found in *Halieaceae* might reflect the spectral properties of sunlight changing with depth or coastal-open ocean transition. Genes for the biosynthesis of retinal, an essential chromophore of rhodopsin, were found only in *A. fuscus* IMCC3088, right next to the PR gene (Fig. S5b) (44). PRs in marine bacteria such as Pelagibacter ubique can enhance survival during nutrient limitation periods (45–47); thus, they may function similarly in *Halieaceae*. The AAP gene cluster found in the “*Ca*. P. aquimaris” JH123 genome showed an arrangement identical to or highly similar to that of closely related HIMB55 and the first gammaproteobacterial AAP strain Congregibacter litoralis KT71^T^ (Fig. S5a) (4).

### Nitrogen and sulfur metabolism.

Genes encoding the enzymes of the complete dissimilatory nitrate reduction to ammonium (DNRA) based on nitrate reductase (*napAB*) and nitrite reductase (*nirBD*) were detected in “*Ca.* L. singularis” IMCC14734, suggesting the potential to retain the transformed nitrogen for biological availability (48). The *napAB* and *nirBD* genes were also identified in “*Ca*. S. marina” IMCC8485 and *H. maricola* IMCC14385, respectively. The catalytic subunit of assimilatory nitrate reductase (*nasA*) was predicted in *H. maricola* IMCC14385. “*Ca.* L. singularis” IMCC14734 had genes for urease and urea transporter (*urtABCDE*), suggesting roles in marine nitrogen cycling.

Two strains in our study had genes for sulfur compound oxidation. The complete sox gene cluster (*soxCDXYZAB*) was identified in *A. fuscus* IMCC3088, indicating the potential of thiosulfate oxidation to sulfate; genes encoding sulfide:quinone oxidoreductase (*sqr*) were detected in *A. fuscus* IMCC3088 and “*Ca*. S. marina” IMCC8485, suggesting that sulfide oxidation could be linked to electron transport chain. The energy conserved from sulfur compound oxidation might contribute to survival under starvation conditions. Taurine dioxygenase was found in all genomes, indicating the putative utilization of taurine, an organosulfur compound abundant in marine environments, as a sulfur and carbon source (49).

### Assimilation of inorganic carbon.

Among many metabolic processes that may utilize light energy harvested by PR and AAP or energy from sulfur oxidation, we focused on carbon fixation or assimilation. None of the six canonical CO_2_ fixation pathways (50, 51) were complete in the six strains, and the recently described reversed oxidative TCA (roTCA) cycle (52, 53) is unlikely to have relevance to the six strains (see the supplemental material for details). Instead, we explored the possibilities that the six strains could reduce CO_2_ to formate, followed by formate assimilation via the reductive glycine pathway (rGlyP) (54–56). First, formate dehydrogenase (EC 1.17.1.9) (FDH), which may function in the reduction of CO_2_ to formate, was found in the genomes of “*Ca*. P. aquimaris” JH123, “*Ca.* S. marina” IMCC8485, and *H. maricola* IMCC14385. Second, in terms of formate assimilation, only three pathways are known to date: the Wood-Ljungdahl (WL) pathway, the serine pathway (57), and the rGlyP. While the WL pathway and serine pathway were incomplete in all six strains, genes for the complete rGlyP were found in “*Ca*. P. aquimaris” JH123, “*Ca.* S. marina” IMCC8485, and *H. maricola* IMCC14385 (54–56). The reduction of formate to methylenetetrahydrofolate (methylene-THF) is predicted to be performed by formate-tetrahydrofolate ligase (*fhs*; present only in the three strains) and methylenetetrahydrofolate dehydrogenase (*folD*). The next steps of the rGlyP leading to pyruvate formation mediated by several genes (*gcvT*, *gcvP*, *lpd*, *gcvH*, *glyA*, and *sdaA*) were also found in “*Ca*. P. aquimaris” JH123, “*Ca.* S. marina” IMCC8485, and *H. maricola* IMCC14385. Thus, these three strains could putatively assimilate CO_2_ by using the rGlyP via the serine route (55, 56). Various carbon fixation pathways can be differentially preferred according to CO_2_ concentrations and available energy (51, 58). The rGlyP represents an optimal balance between efficiency and versatility because of the reduction-first approach in combination with carboxylation and the reaction under various environmental conditions mainly governed by the oxygen sensitivity of formate dehydrogenase; it potentially has an advantage over purely carboxylation pathways such as the Calvin-Benson-Basham (CBB) cycle (59). Given the genomic features indicating overall heterotrophic lifestyles, the rGlyP in these three strains, even if it works, would serve auxiliary roles, which could confer metabolic advantages when organic carbon sources are scarce in oligotrophic marine environments.

In addition, anaplerotic enzymes, which replenish TCA intermediates used for biosynthesis, may facilitate the assimilation of inorganic carbon. PEP carboxykinase (ATP) (*pckA*) and malate dehydrogenase (NADP^+^, decarboxylating; *maeB*) were present in all six strains, while pyruvate carboxylase (*pyc*) was absent in all of them. PEP carboxylase (*ppc*) was absent only in “*Ca.* M. litorale” IMCC11814. The gene encoding Na^+^-translocating oxaloacetate decarboxylase (*oadA*) was detected in all strains except “*Ca*. P. aquimaris” JH123 and “*Ca.* M. litorale” IMCC11814. In marine AAP- or PR-harboring strains, the conversion of light energy into chemical energy by AAP or PR has been shown to allow cells to save carbon necessary for energy generation (respiration) and divert more carbons for assimilation into biomass, which is aided with anaplerotic reactions (60–63). Thus, several anaplerotic enzymes of the six strains in our study may contribute to survival under carbon-depleted but light-replete conditions.

### Polysaccharide degradation.

**FIG 5:**
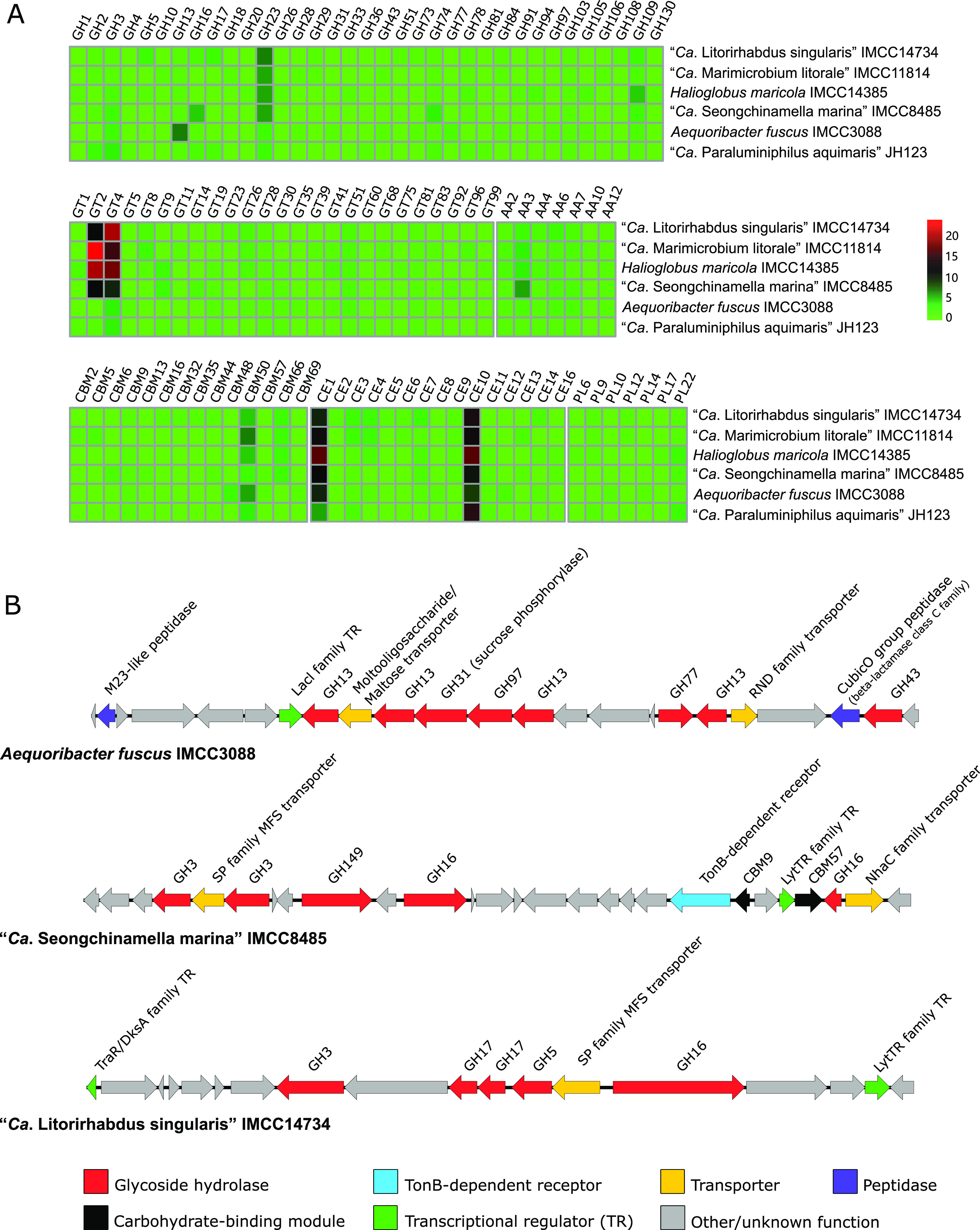
CAZyme gene repertoire of the six strains in this study. (A) Heat map indicating the numbers of genes in each genome assigned to various CAZyme families (GH, glycoside hydrolase; GT, glycosyl transferase; AA, auxiliary carbohydrate-active oxidoreductase; CBM, carbohydrate-binding module; CE, carbohydrate esterase; PL, polysaccharide lyase). Colors in each cell indicate the number of genes based on the legend at the right center. (B) CAZyme gene clusters detected in the genomes of this study. CAZymes, TonB-dependent receptors, transporters, transcriptional regulators and peptidases, and genes involved in other metabolic functions and hypothetical proteins are depicted in different colors based on the legend at the bottom.

Phytoplankton blooms result in the release of large amounts of algal polysaccharides. The decomposition of these polysaccharides requires a diverse repertoire of CAZymes (64). The six strains in this study harbored 75 to 139 genes encoding CAZymes that cover a wide range of classes and families (GH, 37 families; GT, 25; CE, 15; CBM, 14; AA, 7; PL, 7) (Fig. 5A; see details in the supplemental material). In addition, a total of 87 sulfatases were also found, indicating the potential to degrade sulfated polysaccharides (65). Some of the CAZyme families abundant in the six strains were also identified in an alga-associated gammaproteobacterial strain Reinekea forsetii Hel1_31_D35 and flavobacterial *Polaribacter* species (5, 66, 67). These enzymes (e.g., GH23, GH3, CBM50, and CE1) exhibited the highest mean abundance during blooming in the North Sea (22), implicating their important roles in polysaccharide utilization.

Further supporting the potential for polysaccharide degradation, *A. fuscus* IMCC3088, “*Ca.* S. marina” IMCC8485, and “*Ca.* L. singularis” IMCC14734 possessed gene clusters that commonly had CAZymes, transporters, and transcriptional regulators (Fig. 5B; see details in the supplemental material). Although *susC*-*susD* gene pairs, the hallmark of canonical PULs found in *Bacteroidetes*, were not detected in these gene clusters, the gene clusters enriched with CAZymes but devoid of SusC-SusD pairs are regarded to be functional PULs in *Gammaproteobacteria* (24). In comparison with *Bacteroidetes* PULs using PULDB (68), the GH composition of *A. fuscus* IMCC3088 PUL (GH13+GH31+GH97+GH77+GH43) was similar to that of three *Prevotella* species, and the GH composition of “*Ca.* L. singularis” IMCC14734 PUL (GH3+GH17+GH5+GH16) was found in Lutibacter maritimus DSM24450 and *Marinifilaceae* sp. strain SPP2.

### Secondary metabolism.

Prediction of biosynthetic gene clusters (BGCs) by using antiSMASH (69) showed that the six genomes have 2 to 7 BGCs, such as NRPS (non-ribosomal peptide synthetase)-like and RiPP (ribosomally synthesised and post-translationally modified peptide product)-like clusters (Table S4). Although specific metabolites could not be predicted due to low similarity to previously characterized BGCs, the putative potential for secondary metabolite production may provide a competitive advantage to these strains.

### Distribution of the six strains in various oceanic regions.

**FIG 6:**
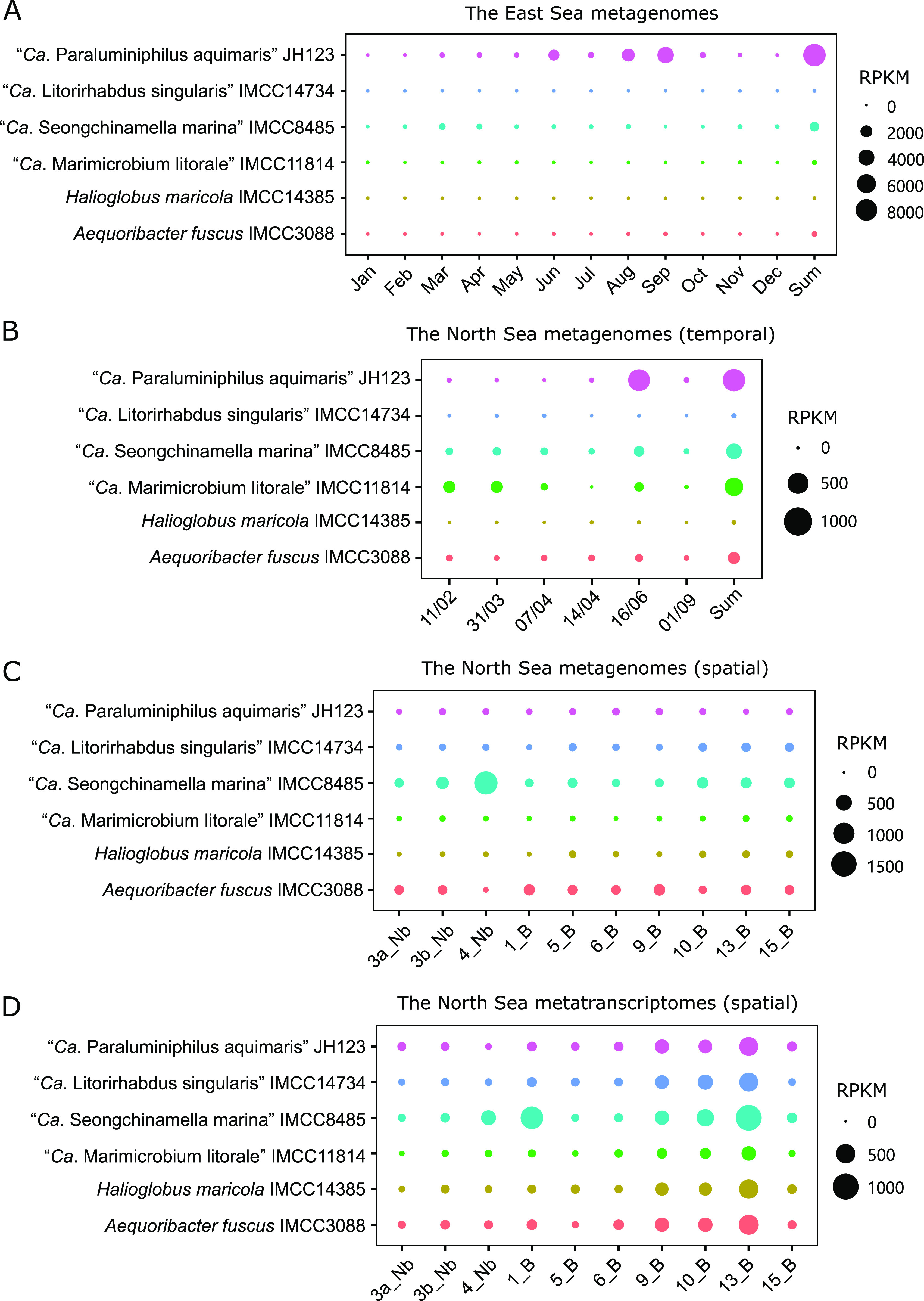
Abundance and transcriptional activities of the six strains in this study. Fragment recruitment analyses were performed using the metagenomes from the time-series samples of the East Sea (A) and the North Sea (B) and the metagenomic (C) and metatranscriptomic (D) data of the North Sea stations. Circle sizes indicate RPKM values (multiplied by 10^6^), based on the legends at the right of each panel. Sample names in panel B indicate sampling dates in the format of day/month. ‘Sum’ columns of panels A and B indicate the sum of RPKM values across months or dates. “Nb” and “B” in the sample names in panels C and D indicate “no-bloom” and “bloom” stations of the North Sea, respectively. Accession numbers of the metagenomes and metatranscriptomes are provided in Table S3 in the supplemental material.

The environmental distribution of the six strains was analyzed by mapping time-series metagenome data from the East Sea and the North Sea to the genomes by using BLASTn, which showed strain-specific temporal variation in abundance. In the East Sea, where five of the six strains of this study were isolated, the highest average abundance was observed in “*Ca.* P. aquimaris” JH123, followed by “*Ca.* S. marina” IMCC8485 (Fig. 6A). “*Ca.* P. aquimaris” JH123 was more abundant from June to September, while “*Ca.* S. marina” IMCC8485 showed higher abundance from March to August. In the North Sea, where omics-based studies on the OM60/NOR5 clade have been actively performed, “*Ca.* P. aquimaris” JH123 showed the highest average abundance, with a peak in June. The next two most abundant strains, “*Ca.* M. litorale” IMCC11814 and “*Ca.* S. marina” IMCC8485, showed the highest abundance in the early spring months in addition to June (Fig. 6B). This strain-specific temporal variation may reflect niche differentiation among the strains.

Read mapping using both the metagenomes and metatranscriptomes obtained from several stations inside and outside the spring bloom in the North Sea (70) revealed a higher transcriptional activity of the six strains in bloom stations (Fig. 6C and D; Fig. S6). The metagenomic data showed that there was not much difference between “bloom” and “no-bloom” stations in the average abundance of all strains, except for that for “*Ca.* S. marina” IMCC8485, which showed noticeably higher abundance in a “no-bloom” station (Fig. S6a). In contrast, in the metatranscriptomic analysis, all strains recruited many more reads in “bloom” stations than in “no-bloom” stations on average (Fig. S6b), suggesting an increase in transcriptional activity in response to blooms. This finding supports the association of the OM60/NOR5 clade (*Halieaceae*) with phytoplankton blooms (5, 22) and also provides an example of a decoupling between abundance and activity in ocean environments (71).

### Metabolic overview of other cultured *Halieaceae* members.

**FIG 7:**
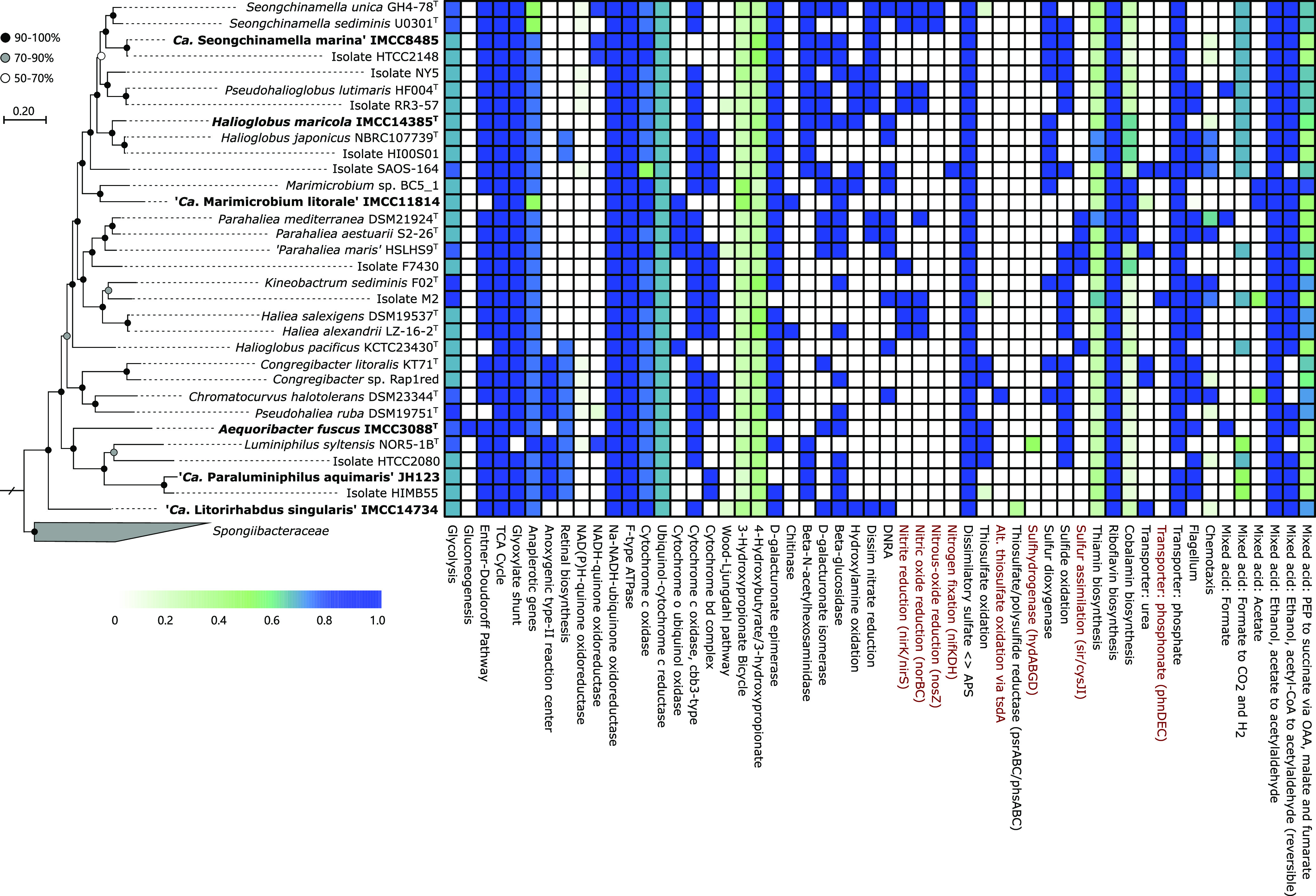
Comparison of the selected metabolic features in cultured *Halieaceae* strains. The phylogenomic tree on the left is from Fig. 2, with the six strains in this study highlighted in bold. The heat map on the right shows the completeness of various metabolic functions and pathways calculated by the KEGG Decoder based on the color scale at the lower left. Functions and pathways not found in any of the six strains of this study but found in some other cultured *Halieaceae* strains are highlighted in red type. PEP, phosphoenolpyruvate; OAA, oxaloacetate.

To further infer the ecological roles of diverse cultured *Halieaceae* strains in marine environments, we investigated various metabolic potentials based on the KEGG database (72) by using BlastKOALA (73), followed by KEGG Decoder (18). Similar to the six strains described here, other members have pathways or genes involved in central carbon metabolism, anaplerotic reactions, oxidative phosphorylation, and light utilization, confirming the aerobic photo- or chemoheterotrophic lifestyles of this family (Fig. 7). In addition to these commonalities, various metabolic potentials not found in the six strains were predicted in other *Halieaceae* genomes. In terms of nitrogen and sulfur metabolism, genes for nitrite reduction (*nirK* and *nirS*), nitric oxide reduction (*norBC*), nitrous oxide reduction (*nosZ*), nitrogen fixation (*nifKDH*), alternative thiosulfate oxidation (via *tsdA*), sulfhydrogenase (*hydABGD*), and sulfur assimilation (*sir* and *cysJI*) were found in some strains. As for phosphorus metabolism, in contrast to the six strains having only a high-affinity phosphate transporter (*pstSCAB*), a phosphonate transporter (*phnDEC*) was found in some other cultured members. Phosphorus availability is a major selective pressure that has shaped the adaptations of abundant bacterial populations (74). Phosphonates, which contain a C–P bond, have been recognized as a potential phosphorus source in phosphate-depleted oligotrophic ecosystems (75). Therefore, *Halieaceae* populations equipped with a phosphonate transporter can effectively compete for phosphorus acquisition under oligotrophic conditions.

### Conclusions.

In this study, we report the cultivation of six pure cultured *Halieaceae* strains and the characterization of their genomes. In addition to the two strains (*H. maricola* IMCC14385 and *A. fuscus* IMCC3088) that were subjected to valid taxonomic descriptions in our previous studies (14, 37), “*Ca.* S. marina” IMCC8485 and “*Ca.* M. litorale” IMCC11814 could be classified as two new species and “*Ca.* P. aquimaris” JH123 and “*Ca.* L. singularis” IMCC14734 could be categorized as species belonging to two new genera of *Halieaceae* based on phylogenetic and genomic analyses, for which the *Candidatus* names were proposed. Metabolic reconstructions revealed mainly aerobic heterotrophic lifestyles, aided by light utilization by PR or AAP, the use of nitrogen and sulfur compounds as alternative electron acceptors and donors, assimilation of inorganic carbon via rGlyP and anaplerotic reactions, and polysaccharide utilization. A metabolic overview of *Halieaceae* further showed versatile metabolic features that may contribute to the ecological success of this family in ocean ecosystems. The novel *Halieaceae* isolates and their genomes obtained in this study could be used in ecophysiological studies to obtain new insights into the function and variability of oceanic microbes.

### Proposal of provisional names for *Halieaceae* strains.

**(i) “*Candidatus* Paraluminiphilus aquimaris” gen. nov., sp. nov., strain JH123.** “*Candidatus* Paraluminiphilus” (Pa.ra.lu.mi.ni’phi.lus. Gr. pref. *para*-like, beside; N.L. masc. n. *Luminiphilus* a bacterial genus; N.L. masc. n. *Paraluminiphilus* like *Luminiphilus*, referring to the close relationship to the genus).

“*Candidatus* Paraluminiphilus aquimaris” (a.qui.ma'ris. L. fem. n. *aqua* water; L. neut. n. *mare* the sea; N.L. gen. n. *aquimaris* of water of the sea).

**(ii) “*Candidatus* Seongchinamella marina” sp. nov. strain IMCC8485.** “*Candidatus* Seongchinamella marina” (ma.ri’na. L. fem. adj. *marina* of the sea, marine).

**(iii) “*Candidatus* Marimicrobium litorale” sp. nov. strain IMCC11814.** “*Candidatus* Marimicrobium litorale” (li.to.ra'le. L. neut. adj. *litorale* of or belonging to the seashore).

**(iv) “*Candidatus* Litorirhabdus singularis” gen. nov., sp. nov., strain IMCC14734.** “*Candidatus* Litorirhabdus” (Li.to.ri.rhab'dus. L. neut. n. *litus-oris*, seashore; N.L. fem. n. *rhabdus* a rod, wand; N.L. fem. n. *Litorirhabdus* a rod that grows in the sea).

“*Candidatus* Litorirhabdus singularis” (sin.gu.la'ris. L. fem. adj. *singularis* alone, singular; referring to the fact that this is a free-living member).

## MATERIALS AND METHODS

### Strain isolation, revival, cultivation, and 16S rRNA gene sequencing.

The overall experimental workflow is shown in Fig. S1 in the supplemental material. All strains were isolated using a standard dilution technique or high-throughput culturing (HTC) based on a dilution-to-extinction method from samples of coastal epipelagic seawaters (depth, 10 or 55 m). For HTC, sample collection, medium preparation, isolation, and cultivation were performed as described by Yang et al. (76), but medium compositions slightly differed between the strains named IMCC and JH (Table S1). DNA extraction, 16S rRNA gene amplification and sequencing, sequence quality inspection, trimming, and phylogenetic analysis were conducted as described by Kang et al. (77). For revival, 200 μL of glycerol stocks from each culture was inoculated into 20 mL of the medium, which was the same as that used for the original isolation. After 4 weeks of incubation at 18°C in the dark, the specimens were stained with 1:2,000 (vol/vol) diluted SYBR green I (Thermo Fisher Scientific, MA, USA), and culture growth was screened using a Guava EasyCyte Plus flow cytometer (Merck Millipore, MA, USA). The purity and identity of the growth-positive cultures (≥5 × 10^4^ cells/mL) were evaluated by amplified 16S rRNA gene sequence chromatography and microscopy. Then, the 16S rRNA gene sequences of all cultured strains and representative clones of *Halieaceae* were used to construct phylogenetic trees based on the neighbor-joining method (78) with Jukes-Cantor correction in MEGA X version 10.1 (79) to preliminarily infer the phylogenetic affiliation of the successfully revived strains (Fig. S2). The sequences were phylogenetically grouped in accordance with previously described methods (1, 3). Six cultures with sustained growth were selected for further analysis based on phylogeny and sequence similarities.

### Colony-forming ability.

The colony-forming ability of the selected cultures was examined by spreading 10 μL of each culture on Marine Agar 2216 (MA; BD Diagnostics), 3M-R2A agar (0.5 g yeast extract, 0.5 g proteose peptone, 0.5 g Casamino Acids, 0.3 g sodium pyruvate, 0.5 g glucose, 0.5 g soluble starch, and 15 g agar) in 1 L of diluted aged seawater (seawater–Milli-Q water = 8:2), 1/10-fold (except for agar) 3M-R2A agar in 1 L of the same diluted aged seawater, 1/10-fold 3M-R2A agar in artificial seawater (ASW; 24.7 g NaCl, 4.7 g MgCl_2_·6H_2_O, 1.4 g CaCl_2_·2H_2_O, 0.7 g KCl, and 6.3 g MgSO_4_·7H_2_O in 1 L of deionized water amended with NH_4_NO_3_, Na_2_HPO_4_·7H_2_O, and NaHCO_3_ at final concentrations of 20 μM, 50 μM, and 2.14 mM, respectively), 3M-R2A agar in diluted ASW (ASW–Milli-Q water = 1:9 [vol/vol]), and 0.5% (wt/vol) peptone, 0.1% (wt/vol) yeast extract, and 1.5% (wt/vol) agar in ASW. The agar plates were aerobically incubated in the dark at 18°C for 1 month. Colonies were transferred several times to fresh plates for purification and stored as glycerol stocks (10%, vol/vol) at −80°C.

### DNA extraction and whole-genome sequencing, genome assembly, and annotation.

Genomic DNA was extracted from stationary-phase cultures by using a DNeasy blood and tissue kit (Qiagen, Hilden, Germany) in accordance with the manufacturer’s protocol with modifications. All cultures were cultivated aerobically at 18°C. For *A. fuscus* IMCC3088 and *H. maricola* IMCC14385, cultures were performed using Erlenmeyer flasks (SPL Life Sciences, Gyeonggi-do, Republic of Korea) and 1/10-fold 3M-R2A broth in 1 L of 80% aged seawater was used. Visible biomass was harvested by centrifugation (12,000 × *g*, 10 min) and divided into several microtubes with an appropriate amount for DNA extraction. For “*Ca.* L. singularis” IMCC14734, “*Ca.* M. litorale” IMCC11814, “*Ca*. S. marina” IMCC8485, and “*Ca.* P. aquimaris” JH123, cells were grown in the same culture medium as that used for the revival experiment (Table S1). After incubation, cells were filtered through filters (Supor-200 membrane disk filters; Pall Gelman Laboratory, Ann Arbor, MI) with a diameter of 47 mm and a pore size of 0.2 μm. Filters with cells were transferred into 5-mL tubes containing 925 μL ATL buffer and 75 μL proteinase K and mixed thoroughly at 56°C for 30 min. Then, the lysate was pipetted into a microtube, combined with 20 μL RNase A, and incubated at 30°C for 20 min. All the reagents used were provided in the DNeasy blood and tissue kit (Qiagen, Hilden, Germany), and the following procedures were performed in accordance with the manufacturer’s protocol. The purity and concentration of DNA samples were examined using a Qubit fluorometer (Life Technologies, Paisley, UK) and a NanoDrop 1000 spectrophotometer (Thermo Scientific, Waltham, MA). The complete genomes of *H. maricola* IMCC14385 and *A. fuscus* IMCC3088 were obtained as previously described (14, 37). Genomes of “*Ca.* L. singularis” IMCC14734, “*Ca.* M. litorale” IMCC11814, “*Ca.* S. marina” IMCC8485, and “*Ca.* P. aquimaris” JH123 were sequenced using an Illumina MiSeq platform (Macrogen, Seoul, Republic of Korea). The quality of the resulting 2 × 300 bp paired-end raw reads was assessed using FastQC (https://www.bioinformatics.babraham.ac.uk/projects/fastqc/). Adaptors, low-quality regions, and short-length sequences were removed using Trimmomatic (version 0.36) (80) with the following parameters: ILLUMINACLIP:TruSeq3-PE-2.fa:2:30:10 LEADING:3 TRAILING:3 SLIDINGWINDOW:4:15 MINLEN:100. *De novo* assembly was conducted using SPAdes (version 3.9.0) in a multicell mode with the option “–careful” and default k-mer values (81). The generated FASTG files were visualized in Bandage (version 0.07) (82) to remove contaminant contigs manually. The contigs longer than 1,000 bp were retained, resulting in more than six contigs for “*Ca.* S. marina” IMCC8485, “*Ca.* M. litorale” IMCC11814, and “*Ca.* L. singularis” IMCC14734 and 3 contigs for “*Ca.* P. aquimaris” JH123. For “*Ca.* P. aquimaris” JH123, another assembly with k-mer lengths of 21, 33, 55, 77, 99, 127, 141, 161, 181, 201, 221, and 241 was established, resulting in a single contig. However, the gap was not successfully filled via PCR by using primers to close the contig putatively because of repeats at the ends. Gene prediction and functional annotation were performed using the JGI-Integrated Microbial Genomes and Microbiomes Expert Review (IMG/MER) system (83) and Prokka (version 1.12) (84). Amino acid biosynthesis was annotated using GapMind (https://papers.genomics.lbl.gov/gaps) (85). Carbohydrate-active enzymes (CAZymes) were detected using the dbCAN webserver against the CAZy database with default settings (86). HMMer (version 3.1) (87) searches with TIGRFAM (profile TIGR04056) and Pfam (profiles PF12771, PF14322, PF12741, PF07980, and PF00884) were used to detect SusC- or SusD-like proteins and sulfatases. Secondary metabolite biosynthetic gene clusters (BGCs) were identified and analyzed using antiSMASH (version 6.1.1) (69).

### Phylogeny.

For the phylogenetic analysis of *Halieaceae* strains and clones, nearly complete 16S rRNA gene sequences were aligned using the SILVA Incremental Aligner (88) and masked with the “ssuref:bacteria” filter as implemented in the ARB software (89). A phylogenetic tree was reconstructed on the basis of the resulting alignment by using RAxML (version 8.2.7) (90) under the GTRGAMMA model with 100 bootstrap replicates. Then, 16S rRNA gene sequence similarities were calculated using the similarity option of the ARB distance matrix program. The genomes of 32 cultured *Halieaceae* strains, the representatives of *Spongiibacteraceae*, and Oleiphilus messinensis ME102^T^ (outgroup) were downloaded from the NCBI GenBank database (Table S2) for phylogenomic analysis based on the concatenated alignment of 92 core genes generated using the UBCG pipeline (version 3.0) (91) with default settings. The final tree was constructed using RAxML with the “PROTGAMMAAUTO” model and 100-bootstrap replications. GTDB-Tk classification was performed to delineate phylogenetic affiliations above the genus level (36). Average nucleotide identities (ANI) and alignment fractions (AF) were calculated using Microbial Species Identifier (MiSI) (92) via the Pairwise ANI tool implemented in the IMG/MER system (https://img.jgi.doe.gov/). Average amino acid identities (AAI) were estimated using the aai.rb script from the enveomics collection (93). Percentages of conserved proteins (POCP) were computed to measure genomic similarities at the amino acid level (35).

For the phylogenetic analysis of the rhodopsins found in the strains, protein sequences similar to the rhodopsin sequences in this study were retrieved from the NCBI RefSeq database by using the BLASTP 2.8.1+ algorithm and aligned using MUSCLE (version 3.8) (94). Phylogenetic trees were constructed via the maximum likelihood method based on the JTT model (95) by using MEGA X (version 10.1) (79) with bootstrap values based on 100 replications. Alignment was visualized with Jalview to predict PR functionality (96).

### Metabolic reconstruction and comparative genomics.

Metabolic pathways were reconstructed on the basis of the KEGG database by using the web tool BlastKOALA (73). For comparative purposes, the genome sequences of previously reported *Halieaceae* strains were downloaded from GenBank and annotated using Prokka before KEGG-based analysis. After the KEGG-based annotation of protein coding genes, assigned KEGG orthologs (KOs) were used to estimate the completeness of selected pathways and functions by using the KEGG Decoder, and a heat map was created using a “static” visualization mode (18). Plots of genomic sequence comparisons were generated with Easyfig (97) or BRIG (98) by using BLASTn.

### Mapping of metagenome and metatranscriptome reads to the genomes.

Metagenome and metatranscriptome reads from diverse surface seawater samples were mapped to the genomes to assess the distribution and activity of the six strains in this study (Table S3). The East Sea metagenome data set was obtained from around a station (38°13′44.8″N, 128°41′6.2″E), where most of our strains were isolated at monthly intervals for 1 year (2009; 12 samples). Briefly, the seawater samples were collected by using a 10-L Niskin bottle at a 10-m depth, kept at 4°C, and brought to a laboratory. From a subsample of 5 L, the microbial biomass was collected on 0.2-μm-pore-size, 47-mm-diameter polyethersulfone membrane filters (Supor; Pall Corp., NY, USA) and stored at −80°C until DNA extraction by using a PowerSoil DNA isolation kit (MoBio, CA, USA). DNA purity and concentration were measured using a Qubit fluorometer and a NanoDrop 1000 spectrophotometer. Metagenomic sequencing data were generated by using the Illumina HiSeq 2500 system at ChunLab, Inc. (Seoul, Republic of Korea). In addition, two public metagenomic sets from the German Bight of the North Sea were used: (i) six metagenomes collected during and after a phytoplankton bloom in 2009 (5) and (ii) 10 metagenomes from stations inside and outside phytoplankton blooms in May 2010 (70). The six metagenomes sequenced using 454 GS FLX Titanium were imported to CLC Genomics Workbench version 5.1 (CLC Bio, Aarhus, Denmark) and trimmed using the following parameters: removal of low-quality sequence, limit of 0.05; removal of ambiguous nucleotides, maximum two nucleotides allowed; and removal of sequences on length, minimum length of 200 nucleotides. Raw metagenomic reads from the Illumina sequencing platform were quality trimmed using Trimmomatic with options “ILLUMINACLIP:TruSeq3-PE-2.fa:2:30:10 LEADING:3 TRAILING:3 SLIDINGWINDOW:4:15 MINLEN:80.” Ten metatranscriptomes from the same North Sea stations were trimmed as described by Voget et al. (99). After rRNA gene regions were masked, the genomic sequences of the strains were used as queries for BLASTn (version 2.10.1+) against the trimmed metagenomes and metatranscriptomes, with the options “-evalue 0.0001 -perc_identity 90 -outfmt 6 -max_target_seqs 1000000 -max_hsps 1.” The numbers of BLAST hits from each pair of genomes and metagenomes/metatranscriptomes were normalized by genome sizes (in kilobase pairs) and metagenome/metatranscriptome sizes (in Mbp), resulting in reads per kilobase per million (RPKM) values. The RPKM values were multiplied by 10^6^ and visualized using the ggplot2 package in R.

### Data availability.

The PCR-generated 16S rRNA gene sequences have been deposited in the GenBank/EMBL/DDBJ database under accession number MT453889 for “*Ca.* P. aquimaris” JH123, MT453888 for “*Ca.* L. singularis” IMCC14374, MT453886 for “*Ca.* M. litorale” IMCC11814, and MT453865 for “*Ca.* S. marina” IMCC8485. The whole-genome sequences have been deposited in the GenBank/EMBL/DDBJ database under accession number CP036501 for “*Ca.* P. aquimaris” JH123, SHNN00000000 for “*Ca.* L. singularis” IMCC14374, SHNO00000000 for “*Ca.* M. litorale” IMCC11814, and SHNP00000000 for “*Ca.* S. marina” IMCC8485. The 12 metagenomes of the East Sea used in this study have been deposited in the GenBank/EMBL/DDBJ database under accession number PRJNA877782.
