# Supplementary material for: Metabolic Versatility of the Family Halieaceae Revealed by the Genomics of Novel Cultured Isolates
Source: Microbiol Spectr. 2023 Mar 14;11(2):e03879-22. doi: 10.1128/spectrum.03879-22 (PMC10100682; doi:10.1128/spectrum.03879-22)
Supplement: Supplemental file 1 — Supplemental material. Download spectrum.03879-22-s0001.pdf, PDF file, 1.6 MB [file spectrum.03879-22-s0001.pdf]

Supplementary Materials

Supplementary Text

Aerobic respiration. Three families of genes encoding the NADH dehydrogenase component of the electron transport chain were detected: (i) the six strains exhibited genes encoding all the canonical subunits (*nqrABCDEF*) of Na^+-translocating NADH-quinone reductase, which functions as a gateway for electrons stepping into the aerobic respiratory chain to establish a sodium gradient for energy coupling (1); (ii) the genes encoding H^+-translocating NADH:ubiquinone reductase comprising up to 14 Nuo subunits were detected only in "*Ca.* S. marina" IMCC8485; (iii) the gene encoding FAD-containing NADH dehydrogenase (*ndh*), a nonproton-translocating enzyme, was detected except for *A. fuscus* IMCC3088 and "*Ca.* S. marina" IMCC8485 (Fig. 3). Regarding complex II, the six strains had genes coding for succinate dehydrogenase (*sdhCDAB*). "*Ca.* L. singularis" IMCC14734 and *H. maricola* IMCC14385 contained genes for fumarate reductase complex (*frdABCD*) although the *frdD* subunit was not explicitly found in *H. maricola* IMCC14385. Fumarate reductase in these strains can be used to generate energy via anaerobic respiration (2). The cytochrome bc_1 complex was identified as complex III in the six strains (Fig. 3).

Assimilation of inorganic carbon. None of the six canonical CO_2 fixation pathways were complete in the six strains. Neither ribulose 1,5-bisphosphate carboxylase/oxygenase (RubisCO) nor phosphoribulokinase, the key enzymes integral to the CO_2 fixation through the Calvin–Benson–Bassham (CBB) cycle, can be detected in the six strains, suggesting that this pathway was missing. Both ATP citrate (pro-S)-lyase and citryl-CoA synthase/citryl-CoA lyase were not annotated, indicating the lack of carbon fixation by the reductive TCA (rTCA) cycle. The Wood–

Ljungdahl (WL) pathway comprises a set of enzymes for reducing CO_2 and producing acetyl-CoA. The absence of acetyl-CoA synthase-CO dehydrogenase and formate dehydrogenase (*fdhA*) suggested that all six strains were incapable of using the WL pathway for acetyl-CoA synthesis. Malonyl-CoA reductase and malyl-CoA lyase functioning as key enzymes of the 3-hydroxypropionate pathway were observed in "*Ca*. P. aquimaris" JH123 and "*Ca.* M. litorale" IMCC11814, respectively. However, the third key enzyme, acrylyl-CoA reductase (NADPH), was not observed, indicating that the 3-hydroxypropionate pathway might not be possible. 3-hydroxypropionate/4-hydroxybutyrate (HP/HB) and dicarboxylate/4-hydroxybutyrate (DC/HB) cycles share a key enzyme 4-hydroxybutyryl-CoA dehydratase (4-BUDH) (3), which was identified in "*Ca*. P. aquimaris" JH123, *A. fuscus* IMCC3088, and "*Ca.* S. marina" IMCC8485. No other function for this gene can be assigned other than the conversion of 4-hydroxybutyryl-CoA to crotonyl-CoA, suggesting that 4-BUDHs could be presumed to be functional. However, other genes involved specifically in the HP/HB and DC/HB cycles were not completely found. Recently, Garritano et al. claimed that all genes for the HP/HB cycle were found in MAGs assigned to the genus *Luminiphilus*, which belongs to the family *Halieaceae* and is closely related to a few strains of this study (4). Prompted by this finding, we checked the IMG annotation of the genome of *L. syltensis* NOR5-1B^T, the only isolate of the genus *Luminiphilus* that has been validly published. Further, the two isolates (HTCC2080 and HIMB5), which are closely related with *L. syltensis*, were also checked. Based on the KEGG pathway reconstruction as implemented in the IMG database, however, all the three genomes were found to lack many genes for the HP/HB cycle.

Recently, carbon fixation through the reversed oxidative TCA (roTCA) cycle has been found in a few anaerobic thermophiles (5-7). The roTCA cycle cannot be found bioinformatically

because all steps are sharing enzymes with the oxidative TCA cycle that use 2-oxoglutarate:ferredoxin oxidoreductase for oxidation of 2-oxoglutarate. Therefore, many prokaryotes that have the TCA cycle equipped with 2-oxoglutarate:ferredoxin oxidoreductase can be regarded to be capable of carbon fixation via the roTCA cycle at least theoretically, which is the case for the five strains of this study. Note that the strain JH123 does not encode 2-oxoglutarate:ferredoxin oxidoreductase. But, the roTCA cycle is unlikely to have physiological relevance to the five strains because the cycle has been demonstrated to be functional only under thermophilic, strictly anaerobic, and high-CO_2 (20%) growth conditions.

Other carbon metabolism. The six strains differed in genes for the degradation of fructose, lactose, galactan, melibiose, stachyose, raffinose, galactose, sucrose, and cellulose (Fig. 3). Among them, "*Ca.* P. aquimaris" JH123 had the potential to degrade all these substrates except cellulose. Furthermore, the genes for the degradation of galactose and cellulose were found in "*Ca.* M. litorale" IMCC11814 and *H. maricola* IMCC14385, respectively; the genes for the degradation of sucrose and fructose were detected in *A. fuscus* IMCC3088. The genes for ribose/D-xylose transporter (*rbsBCA*) were identified in *H. maricola* IMCC14385, conferring the ability for monosaccharide uptake. The resulting differences in substrate preferences might lead to niche separation and spatiotemporal dynamics of diverse *Halieaceae* lineages. Some strains retained pathways for the biosynthesis of storage polymers, such as glycogen (*A. fuscus* IMCC3088) and poly-*β*-hydroxybutyrate ("*Ca.* S. marina" IMCC8485 and *H. maricola* IMCC14385), which might facilitate survival under carbon-limiting conditions.

Multiple genes encoding enzymes involved in pyruvate fermentation were detected in the genomes (Fig. 3). The gene encoding pyruvate:ferredoxin oxidoreductase (Por, *por*), which converts pyruvate and CoA into CO_2 and acetyl-CoA, was detected in *H. maricola* IMCC14385

and "*Ca.* L. singularis" IMCC14734. Four copies of the genes for pyruvate formate lyase (Pfl, *pflD*), which converts pyruvate and CoA into formate and acetyl-CoA, were detected in *A. fuscus* IMCC3088. Pfl is known to be essential for various anaerobic metabolisms, including pyruvate fermentation, in diverse bacteria (8-10). The genes encoding Pfl in *A. fuscus* IMCC3088 were located within genomic islands as predicted by IslandViewer 4, indicating that horizontal gene transfer could not be excluded (11). Furthermore, genes for acetaldehyde dehydrogenase (acetylating) and alcohol dehydrogenase, which convert acetyl-CoA to ethanol via acetaldehyde, were found in the six strains. Taken together, the potential of pyruvate fermentation to ethanol was found in *H. maricola* IMCC14385, "*Ca.* L. singularis" IMCC14734 (via Por), and *A. fuscus* IMCC3088 (via Pfl). The gene encoding 2-oxoacid:ferredoxin oxidoreductase (OFOR), which could convert pyruvate to acetyl-CoA in some anaerobic bacteria (12), was detected except for "*Ca.* P. aquimaris" JH123, implying that the two more strains ("*Ca.* S. marina" IMCC8485 and "*Ca.* M. litorale" IMCC11814) might also perform pyruvate fermentation.

Polysaccharide degradation. GHs were dominant in diversity but moderate in gene frequency (154 genes) of all CAZymes, with the highest frequencies of GH23 (31 genes), GH109 (16 genes), and GH3 (14 genes). Furthermore, the six strains possessed GTs with the highest frequencies of GT4 (72 genes) and GT2 (69 genes), CEs with the highest frequencies of CE10 (79 genes) and CE1 (68 genes), and AAs and PLs with the highest frequencies of AA3 (20 genes) and PL22 (11 genes), respectively (Fig. 5a).

Among PULs (Fig. 5b), "*Ca.* L. singularis" IMCC14734 had the simplest one, with five GHs, a sugar porter family MFS transporter, and two transcriptional regulators (TraR/DksA and LytTR families); *A. fuscus* IMCC3088 gene cluster had eight GHs, two transporters (maltose/maltooligosaccharide transporter and RND family efflux transporter MFP subunit), and

a LacI family transcriptional regulator; "*Ca.* S. marina" IMCC8485 had a gene cluster composed of five GHs, two transporters (SP family MFS and NhaC family), and a LytTR family transcriptional regulator. *A. fuscus* IMCC3088 contained M23-like and CubicO peptidases, while "*Ca.* S. marina" IMCC8485 contained two CBMs and a TonB-dependent receptor (TBDR). The presence of transporters and/or TBDR in PULs has been suggested to be analogous to canonical PULs equipped with SusC/SusD (13). For *A. fuscus* IMCC3088, the coupling of peptidases and CAZymes for the degradation of proteins and carbohydrates, respectively, could lead to its rapid growth, as shown in alga-associated *Formosa* sp. Hel1_33_131, which also had a small genome (2.7 Mbp) (14).

Amino acid, vitamin, and nucleotide biosynthesis. Among the 20 standard amino acids, genes related to alanine biosynthesis were absent from the six strains; this finding does not mean alanine auxotrophy, but it is highly likely caused by challenging annotation of nonspecific transaminases (15). The six strains were found to be auxotrophic for two vitamin B compounds: thiamine (vitamin B1) and adenosylcobalamin (vitamin B12). By contrast, the strains harbored genes for the biosynthesis of riboflavin (vitamin B2), pyridoxal-5-phosphate (vitamin B6), pantothenate (vitamin B5), nicotinamide adenine dinucleotide (NAD; vitamin B3), and folate (vitamin B9) (Fig. 3). Biotin (vitamin B7) biosynthesis was complete in only four genomes, with *A. fuscus* IMCC3088 and "*Ca.* P. aquimaris" JH123 missing essential genes. All the six strains had the complete gene sets for the *de novo* biosynthesis of purine and pyrimidine nucleotides except for a gene encoding 5-(carboxyamino) imidazole ribonucleotide synthase (*purK*) that was not predicted in "*Ca.* S. marina" IMCC8485 and "*Ca.* M. litorale" IMCC11814.

Supplementary Tables and Figures

Table S1. Media composition for initial isolation and revival experiment.

Components	Compounds	Final concentration (IMCC)	Final concentration (JH)
Ammonium	NH_4Cl	10 μM	10 μM
Phosphate	KH_2PO_4	10 μM	10 μM
Carbon mixture	Glucose	50 μM	50 μM
	Sodium pyruvate	100 μM	100 μM
	Ribose	50 μM	50 μM
	N-acetyl-D-glucosamine	50 μM	50 μM
	Glycerol	50 μM	50 μM
	Methylamine	50 μM	50 μM
	Acetate	50 μM	Not added
Vitamin mixture	Thiamine·HCl	59 nM	59 nM
	Niacin	81 nM	81 nM
	Ca-Pantothenate	84 nM	84 nM
	Pyridoxine	59 nM	59 nM
	Biotin	409 pM	409 pM
	Folic acid	453 pM	453 pM
	Vitamin B_{12}	70 pM	70 pM
	Myo-inositol	555 nM	555 nM
	p-Aminobenzoic acid	7 nM	7 nM
Trace metal	$FeCl_3 \cdot 6H_2O$	117 nM	117 nM
	$MnCl_2 \cdot 4H_2O$	9 nM	9 nM
	$ZnSO_4 \cdot 7H_2O$	800 pM	800 pM
	$CoCl_2 \cdot 6H_2O$	500 pM	500 pM
	$Na_2MoO_4 \cdot 2H_2O$	300 pM	300 pM
	Na_2SeO_3	1 nM	1 nM
	$NiCl_2 \cdot 6H_2O$	1 nM	1 nM
Catalase		Not added	10 U mL^{-1}
Amino acid mixture	20 standard amino acids	Not added	100 nM for each

Table S2. Genome sequences of the cultured members of the family *Halieaceae* and related strains used for the reconstruction of phylogenomic trees.

Strain No.	GenBank	Contigs	Taxonomy
Aequoribacter fuscus IMCC3088[T]	CP036423	1	*Halieaceae*
"*Ca*. Litorirhabdus singularis" IMCC14734	SHNN00000000	20	*Halieaceae*
"*Ca*. Marimicrobium litorale" IMCC11814	SHNO00000000	9	*Halieaceae*
"*Ca*. Seongchinamella marina" IMCC8485	SHNP00000000	22	*Halieaceae*
Halioglobus maricola IMCC14385[T]	CP036422	1	*Halieaceae*
"*Ca*. Paraluminiphilus aquimaris" JH123	CP036501	1	*Halieaceae*
Congregibacter litoralis KT71[T]	CM002299	1	*Halieaceae*
Congregibacter sp. Rap1red	ACCX00000000	29	*Halieaceae*
Haliea salexigens DSM 19537[T]	AUHJ00000000	78	*Halieaceae*
Halioglobus sp. HI00S01	LWEE00000000	415	*Halieaceae*
HIMB55	AGIF00000000	1	*Halieaceae*
HTCC2080	AAVV00000000	25	*Halieaceae*
HTCC2148	ABXQ00000000	75	*Halieaceae*
Luminiphilus syltensis NOR5-1B[T]	ACCY00000000	1	*Halieaceae*
Pseudohaliea rubra DSM 19751[T]	AUVB01000000	90	*Halieaceae*
Halioglobus japonicus NBRC 107739[T]	CP019450	1	*Halieaceae*
RR3-57	CP019046	1	*Halieaceae*
Pseudohalioglobus lutimaris HF004[T]	PKUS00000000	79	*Halieaceae*
Chromatocurvus halotolerans DSM 23344[T]	QQSW00000000	68	*Halieaceae*
Kineobactrum sediminis F02[T]	PKLZ00000000	39	*Halieaceae*
Parahaliea mediterranea DSM 21924[T]	QEWO00000000	29	*Halieaceae*
Seongchinamella sediminis U0301[T]	QRAN00000000	72	*Halieaceae*
Haliea alexandrii LZ-16-2[T]	RFLW00000000	12	*Halieaceae*
Parahaliea aestuarii S2-26[T]	VRYZ00000000	23	*Halieaceae*
Seongchinamella unica GH4-78[T]	SMSE00000000	10	*Halieaceae*
Parahaliea maris HSLHS9[T]	VRZA00000000	23	*Halieaceae*
SAOS-164	SRLE00000000	66	*Halieaceae*
M2	CP048711	1	*Halieaceae*
BC5_1	CACSIL000000000	53	*Halieaceae*
Halioglobus pacificus KCTC23430[T]	BMYM00000000	8	*Halieaceae*
NY5	VTUX00000000	23	*Halieaceae*
F7430	JACFXU000000000	18	*Halieaceae*
Oceanicoccus sagamiensis NBRC 107125[T]	CP019343	1	*Spongiibacteraceae*
Dasania marina DSM 21967[T]	ARDZ00000000	20	*Spongiibacteraceae*
Spongiibacter tropicus DSM 19543[T]	ATUS00000000	7	*Spongiibacteraceae*
Zhongshania marina DSW25-10[T]	RHGB0000000	46	*Spongiibacteraceae*
Zhongshania marina ZX-21[T]	PQGG00000000	46	*Spongiibacteraceae*
Zhongshania aliphaticivorans SM2[T]	CP014544	1	*Spongiibacteraceae*
Spongiibacter marinus DSM 19753[T]	AULP00000000	22	*Spongiibacteraceae*
BDW918	AJMK00000000	81	*Spongiibacteraceae*
Spongiibacter sp. IMCC21906	CP011477	1	*Spongiibacteraceae*
HTCC2143	AAVT00000000	30	*Spongiibacteraceae*
KOV_DT_Chl	FQLF00000000	7	*Spongiibacteraceae*
Oleiphilus messinensis ME102[T]	CP021425	1	*Oleiphilaceae*

Table S3. Metagenomes and metatranscriptomes from free-living bacterioplankton that were used in this study.

Sampling sites	Samples	Year	Size fraction	Platform	SRA run ID	Library
**Metagenomes**						
East Sea	Jan	2009	≥0.2 µm	Illumina HiSeq 2500	SRR21474786	Paired
East Sea	Feb	2009	≥0.2 µm	Illumina HiSeq 2500	SRR21474785	Paired
East Sea	Mar	2009	≥0.2 µm	Illumina HiSeq 2500	SRR21474782	Paired
East Sea	Apr	2009	≥0.2 µm	Illumina HiSeq 2500	SRR21474781	Paired
East Sea	May	2009	≥0.2 µm	Illumina HiSeq 2500	SRR21474780	Paired
East Sea	Jun	2009	≥0.2 µm	Illumina HiSeq 2500	SRR21474779	Paired
East Sea	Jul	2009	≥0.2 µm	Illumina HiSeq 2500	SRR21474778	Paired
East Sea	Aug	2009	≥0.2 µm	Illumina HiSeq 2500	SRR21474777	Paired
East Sea	Sep	2009	≥0.2 µm	Illumina HiSeq 2500	SRR21474776	Paired
East Sea	Oct	2009	≥0.2 µm	Illumina HiSeq 2500	SRR21474775	Paired
East Sea	Nov	2009	≥0.2 µm	Illumina HiSeq 2500	SRR21474784	Paired
East Sea	Dec	2009	≥0.2 µm	Illumina HiSeq 2500	SRR21474783	Paired
**Metagenomes**						
North Sea	11/02	2009	0.2-3 µm	454 GS FLX	ERR091527	Single
North Sea	31/03	2009	0.2-3 µm	454 GS FLX	ERR091532	Single
North Sea	07/04	2009	0.2-3 µm	454 GS FLX	ERR091533	Single
North Sea	14/04	2009	0.2-3 µm	454 GS FLX	ERR091538	Single
North Sea	16/06	2009	0.2-3 µm	454 GS FLX	ERR091553	Single
North Sea	01/09	2009	0.2-3 µm	454 GS FLX	ERR091555	Single
**Metagenomes** (Nb, No bloom; B, Bloom)						
North Sea	3a_Nb	2010	0.2-2.7 µm	Illumina GIIA	SRR2094816	Paired
North Sea	3b_Nb	2010	0.2-2.7 µm	Illumina GIIA	SRR2094823	Paired
North Sea	4_Nb	2010	0.2-2.7 µm	Illumina GIIA	SRR2094826	Paired
North Sea	1_B	2010	0.2-2.7 µm	Illumina GIIA	SRR2094812	Paired
North Sea	5_B	2010	0.2-2.7 µm	Illumina GIIA	SRR2094830	Paired
North Sea	6_B	2010	0.2-2.7 µm	Illumina GIIA	SRR2094839	Paired
North Sea	9_B	2010	0.2-2.7 µm	Illumina GIIA	SRR2094844	Paired
North Sea	10_B	2010	0.2-2.7 µm	Illumina GIIA	SRR2094848	Paired
North Sea	13_B	2010	0.2-2.7 µm	Illumina GIIA	SRR2094852	Paired
North Sea	15_B	2010	0.2-2.7 µm	Illumina GIIA	SRR2094855	Paired
**Metatranscriptomes** (Nb, No bloom; B, Bloom)						
North Sea	3a_Nb	2010	0.2-2.7 µm	Illumina GIIA	SRR2094818	Single
North Sea	3b_Nb	2010	0.2-2.7 µm	Illumina GIIA	SRR2094819	Single
North Sea	4_Nb	2010	0.2-2.7 µm	Illumina GIIA	SRR2094824	Single
North Sea	1_B	2010	0.2-2.7 µm	Illumina GIIA	SRR2094813	Single
North Sea	5_B	2010	0.2-2.7 µm	Illumina GIIA	SRR2094827	Single
North Sea	6_B	2010	0.2-2.7 µm	Illumina GIIA	SRR2094834	Single
North Sea	9_B	2010	0.2-2.7 µm	Illumina GIIA	SRR2094841	Single
North Sea	10_B	2010	0.2-2.7 µm	Illumina GIIA	SRR2094847	Single
North Sea	13_B	2010	0.2-2.7 µm	Illumina GIIA	SRR2094851	Single
North Sea	15_B	2010	0.2-2.7 µm	Illumina GIIA	SRR2094854	Single

Table S4. Biosynthetic gene clusters (BGCs) in the genomes of the six strains of this study as predicted by antiSMASH (v6.1.1)

BGC type*	JH123	IMC3088	IMC8485	IMC14385	IMC11814	IMC14734
NRPS-like			2	1		
RiPP-like			1	2	1	1
RRE-containing			1	2	1	2
Betalactone	1	1	1	1		
Hserlactone						1
Lassopeptide			1	1		
Terpene	1	1				

*Abbreviations: NRPS-like, non-ribosomal peptide synthetase cluster-like fragment; RiPP-like, other unspecified ribosomally synthesized and post-translationally modified peptide product (RiPP) cluster; RRE-containing, RRE-element containing cluster; Betalactone, beta-lactone containing protease inhibitor; Hserlactone, homoserine lactone cluster; Lassopeptide, Lasso peptide cluster.

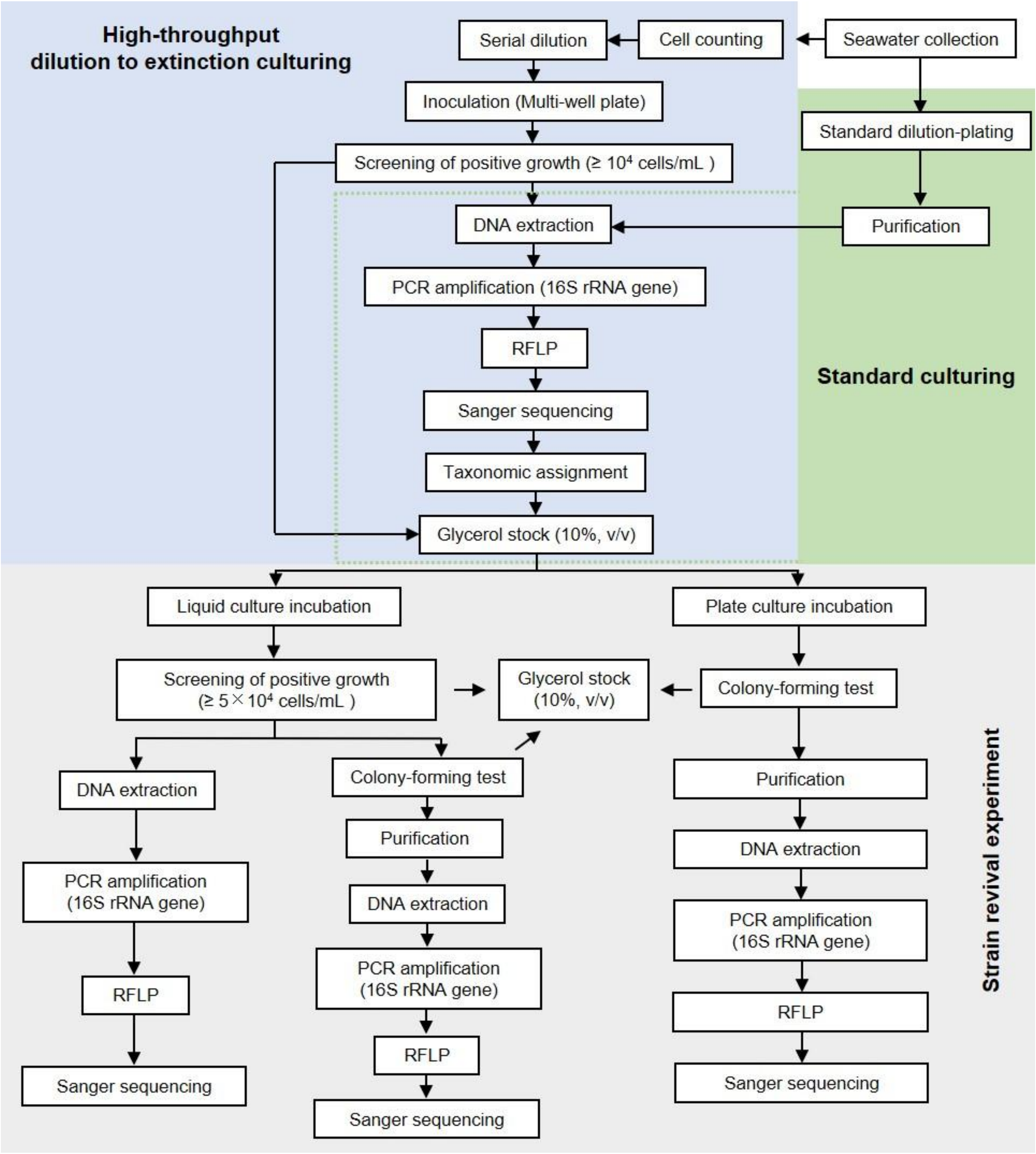

Fig. S1. The experimental workflow of strain revival in this study. All the isolates from dilution-to-extinction culturing and standard dilution-plating methods stored in glycerol stocks were used for reviving tests. Individual pure cultures with positive growth were phylogenetically analyzed.

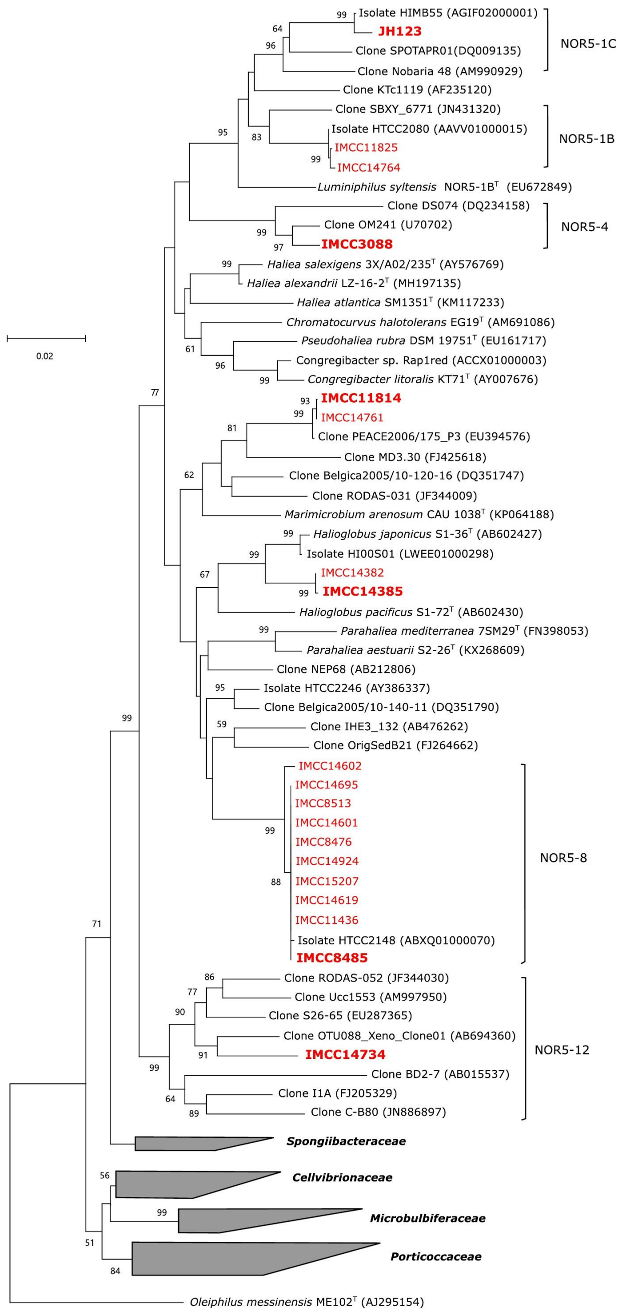

Fig. S2. Phylogenetic placement of the successfully revived cultures within the *Halieaceae* members based on 16S rRNA gene sequences. The phylogenetic tree was reconstructed under the neighbor-joining criterion and rooted using the 16S rRNA gene sequence of *Oleiphilus messinensis* ME102^{T} (AJ295154). All successfully revived *Halieaceae* strains are indicated in red. The six strains selected for genome sequencing are indicated in red and bold. Subclades were designated following the scheme proposed by (7) and indicated by red brackets. Bootstrap values of 50% or above (percentages of 1000 bootstrap resamplings) were indicated. Scale bar, 0.02 substitutions per nucleotide position.

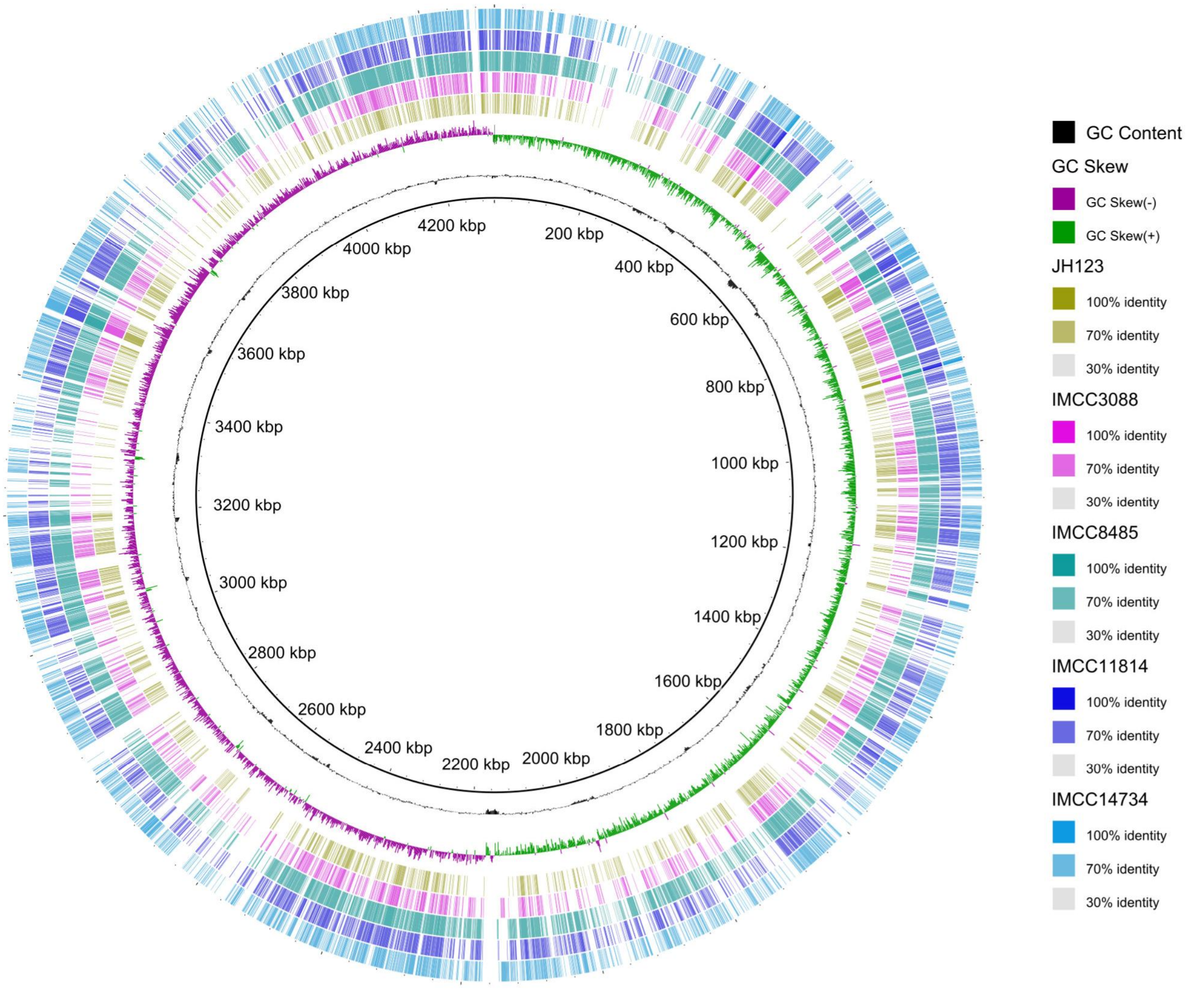

Fig. S3. Genome comparison of the six strains with reference to the *H. maricola* IMCC14385 genome. Innermost rings indicate G+C content (black) and GC skew (purple/green) of the reference genome. The remaining colored rings show BLASTn-based sequence identity (>30%) between each genome and the reference. The graphical view was generated using BRIG (BLAST Ring Image Generator) after performing a BLASTn analysis.

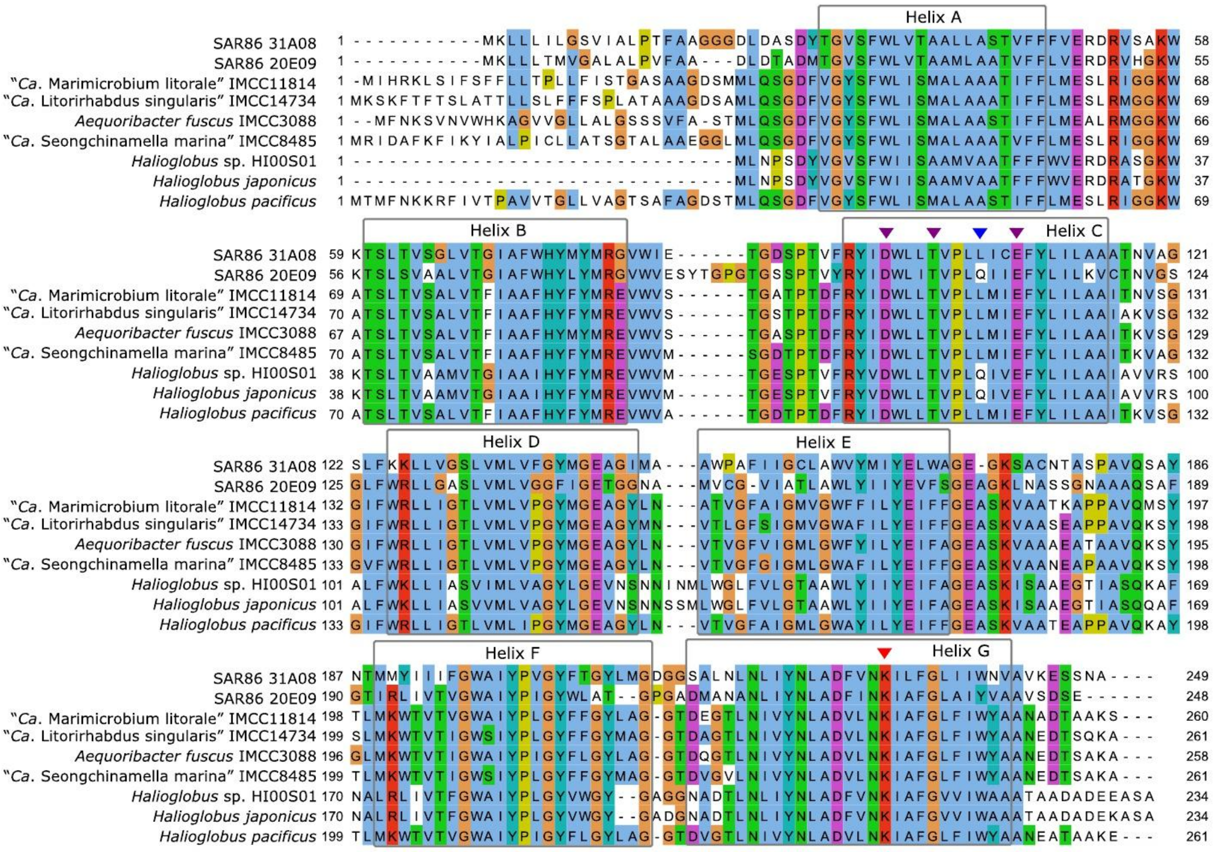

Fig. S4. Multiple alignments of the predicted amino acid sequences of PR of the cultured *Halieaceae* strains and the SAR86 members of *Gammaproteobacteria*. Numbers on the right indicate amino acid residue numbers. The arrows indicate the positions of the key residues for PR functionality: purple, position 97 (Asp, D), 101 (Thr, T) and 108 (Glu, E) for proton pumping; blue, position 105 (Gln, Q for blue or Leu, L for green) for spectral tuning; red, the position of lysine (Lys, K) residue for covalent attachment of retinal to the opsin. Note that amino acid numbering followed the PR sequences of the SAR86 at the top (SAR86 31A08). Boxes with solid lines show predicted transmembrane regions. Amino acid sequences were aligned using MUSCLE version 3.8. Residues are colored according to the "Clustal" colorcode (http://www.jalview.org/help/html/colourSchemes/clustal.html).

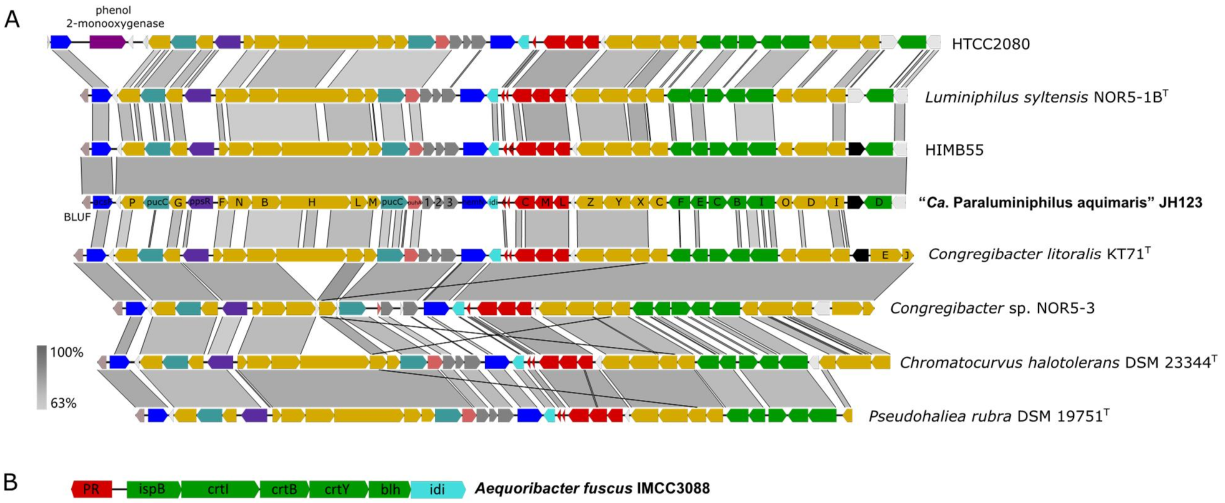

Fig. S5. Gene maps showing the genomic regions around photoheterotrophy-related genes found in cultured *Halieaceae* strains. A, Comparison of AAP gene clusters between the "*Ca*. P. aquimaris" JH123 genome and other *Halieaceae* genomes. The arrows indicate the direction of transcription. Genes are colored according to biological categories: yellow, *bch* genes; green, *crt* genes; red, *puf* genes; blue, *heme* genes; grey, additional conserved genes; white, hypothetical genes. Similar regions were connected by grey rectangles indicating BLASTn-based similarity following the scale bar in the lower left. B, Organization of genes for PR and retinal biosynthesis identified in *A. fuscus* IMCC3088.

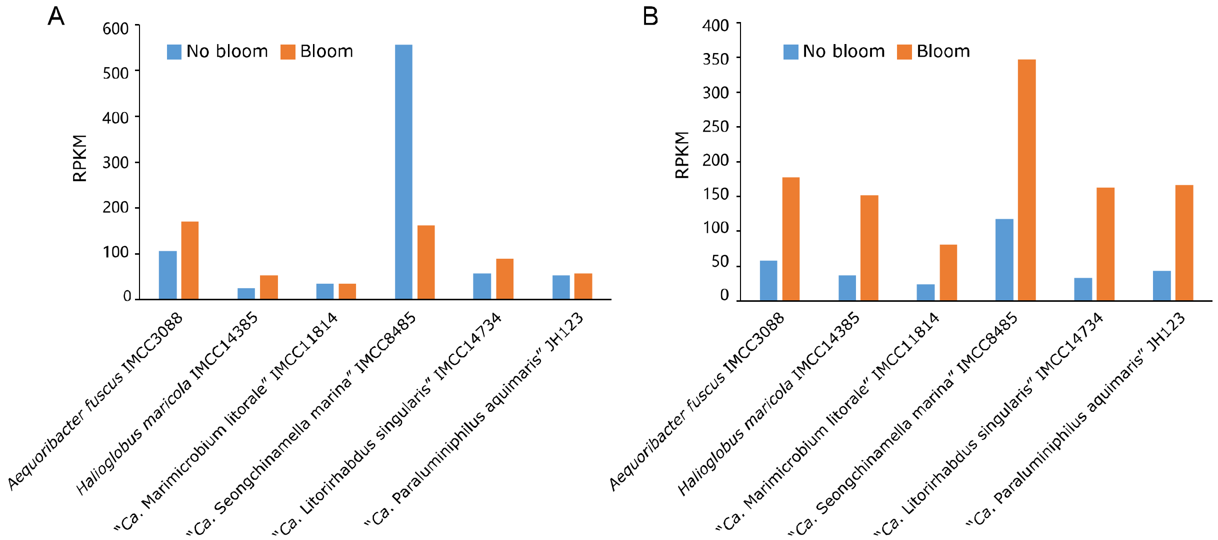

Fig. S6. Comparisons of mean RPKM of the six strains between 'bloom' and 'no bloom' stations in the North Sea. Reads per kilobase of genome per megabase (RPKM) of metagenome (A) or metatranscriptome (B) was calculated based on fragment recruitment analysis using the same samples as shown in Fig. 5c and d.

References

1. Barquera B. 2014. The sodium pumping NADH:quinone oxidoreductase (Na^+-NQR), a unique redox-driven ion pump. J Bioenerg Biomembr 46:289–298.
2. Cecchini G, Schröder I, Gunsalus RP, Maklashina E. 2002. Succinate dehydrogenase and fumarate reductase from *Escherichia coli*. Biochim Biophys Acta Bioenerg 1553:140–157.
3. Berg IA, Kockelkorn D, Ramos-Vera WH, Say RF, Zarzycki J, Hügler M, Alber BE, Fuchs G. 2010. Autotrophic carbon fixation in archaea. Nat Rev Microbiol 8:447–460.
4. Garritano AN, Song W, Thomas T. 2022. Carbon fixation pathways across the bacterial and archaeal tree of life. PNAS Nexus 1:pgac226. https://doi.org/10.1093/pnasnexus/pgac226.
5. Mall A, Sobotta J, Huber C, Tschirner C, Kowarschik S, Bačnik K, Mergelsberg M, Boll M, Hügler M, Eisenreich W, Berg IA. 2018. Reversibility of citrate synthase allows autotrophic growth of a thermophilic bacterium. Science 359:563–567.
6. Nunoura T, Chikaraishi Y, Izaki R, Suwa T, Sato T, Harada T, Mori K, Kato Y, Miyazaki M, Shimamura S, Yanagawa K, Shuto A, Ohkouchi N, Fujita N, Takaki Y, Atomi H, Takai K. 2018. A primordial and reversible TCA cycle in a facultatively chemolithoautotrophic thermophile. Science 359:559–563.
7. Steffens L, Pettinato E, Steiner TM, Mall A, König S, Eisenreich W, Berg IA. 2021. High CO_2 levels drive the TCA cycle backwards towards autotrophy. Nature 592:784–788.
8. Hasona A, Kim Y, Healy FG, Ingram LO, Shanmugam KT. 2004. Pyruvate formate lyase and acetate kinase are essential for anaerobic growth of *Escherichia coli* on xylose. J Bacteriol 186:7593–7600.
9. Doi Y, Ikegami Y. 2014. Pyruvate formate-lyase is essential for fumarate-independent anaerobic glycerol utilization in the *Enterococcus faecalis* strain W11. J Bacteriol 196:2472–2480.
10. Flynn CM, Hunt KA, Gralnick JA, Srienc F. 2012. Construction and elementary mode analysis of a metabolic model for *Shewanella oneidensis* MR-1. Biosystems 107:120–128.
11. Bertelli C, Laird MR, Williams KP, Lau BY, Hoad G, Winsor GL, Brinkman FSL. 2017. IslandViewer 4: expanded prediction of genomic islands for larger-scale datasets. Nucleic Acids Res 45:W30–W35.
12. Fukuda E, Wakagi T. 2002. Substrate recognition by 2-oxoacid:ferredoxin oxidoreductase from *Sulfolobus* sp. strain 7. Biochim Biophys Acta Prot Struct Mol Enzym 1597:74–80.

13. Grondin JM, Tamura K, Déjean G, Abbott DW, Brumer H. 2017. Polysaccharide utilization loci: fueling microbial communities. J Bacteriol 199:e00860-16.

14. Unfried F, Becker S, Robb CS, Hehemann J-H, Markert S, Heiden SE, Hinzke T, Becher D, Reintjes G, Krüger K, Avcı B, Kappelmann L, Hahnke RL, Fischer T, Harder J, Teeling H, Fuchs B, Barbeyron T, Amann RI, Schweder T. 2018. Adaptive mechanisms that provide competitive advantages to marine bacteroidetes during microalgal blooms. Isme J 12:2894–2906.

15. Price MN, Deutschbauer AM, Arkin AP. 2020. GapMind: automated annotation of amino acid biosynthesis. mSystems 5:e00291-20.
